# Epigenetic and Genetic Factors Related to Curve Progression in Adolescent Idiopathic Scoliosis: A Systematic Scoping Review of the Current Literature

**DOI:** 10.3390/ijms23115914

**Published:** 2022-05-25

**Authors:** Cesare Faldini, Marco Manzetti, Simona Neri, Francesca Barile, Giovanni Viroli, Giuseppe Geraci, Francesco Ursini, Alberto Ruffilli

**Affiliations:** 1 Department of Biomedical and Neuromotor Science-DIBINEM, 1st Orthopaedic and Traumatologic Clinic, IRCCS Istituto Ortopedico Rizzoli, University of Bologna, Via Giulio Cesare Pupilli 1, 40136 Bologna, Italy; cesare.faldini@ior.it (C.F.); marco.manzetti@ior.it (M.M.); francesca.barile@ior.it (F.B.); giovanni.viroli@ior.it (G.V.); giuseppe.geraci@ior.it (G.G.); alberto.ruffilli@ior.it (A.R.); 2 Medicine and Rheumatology Unit, IRCCS Istituto Ortopedico Rizzoli, Via Giulio Cesare Pupilli 1, 40136 Bologna, Italy; francesco.ursini@ior.it; 3 Department of Biomedical and Neuromotor Science—DIBINEM, University of Bologna, Via Giulio Cesare Pupilli 1, 40136 Bologna, Italy

**Keywords:** epigenetics, genetics, adolescent idiopathic scoliosis, curve progression, prognostic

## Abstract

Adolescent idiopathic scoliosis (AIS) is a progressive deformity of the spine. Scoliotic curves progress until skeletal maturity leading, in rare cases, to a severe deformity. While the Cobb angle is a straightforward tool in initial curve magnitude measurement, assessing the risk of curve progression at the time of diagnosis may be more challenging. Epigenetic and genetic markers are potential prognostic tools to predict curve progression. The aim of this study is to review the available literature regarding the epigenetic and genetic factors associated with the risk of AIS curve progression. This review was carried out in accordance with Preferential Reporting Items for Systematic Reviews and Meta-analyses (PRISMA) guidelines. The search was carried out in January 2022. Only peer-reviewed articles were considered for inclusion. Forty studies were included; fifteen genes were reported as having SNPs with significant association with progressive AIS, but none showed sufficient power to sustain clinical applications. In contrast, nine studies reporting epigenetic modifications showed promising results in terms of reliable markers. Prognostic testing for AIS has the potential to significantly modify disease management. Most recent evidence suggests epigenetics as a more promising field for the identification of factors associated with AIS progression, offering a rationale for further investigation in this field.

## 1. Introduction

Adolescent Idiopathic Scoliosis (AIS) is a complex three-dimensional deformity of the spine, with a different grade of involvement of the frontal, sagittal, and axial planes [1]. It affects 2–3% of the adolescent population [2]; females are more often involved than males [3].

The diagnosis of scoliosis is based on patient clinical examination and radiographical evaluation [4]. After AIS is diagnosed, patients need different management (ranging from observation alone to orthotic treatment and surgical correction) according to curve magnitude at the time of diagnosis and curve progression potential.

Scoliotic curves progress until skeletal maturity, causing important aesthetic problems, such as humps, with psychological problems and loss of self-esteem, coronal, and/or sagittal imbalance and muscle fatigue [5]. In rare cases, the curve progression can lead to a severe deformity with the occurrence of a lung restrictive disease, a consequent increase in right atrial and ventricular pressure, alongside neurological impairment [6].

While the Cobb angle is a straightforward tool in initial curve magnitude measurement, assessing the risk of curve progression for each patient at the time of diagnosis may be more challenging.

**Figure 1:**
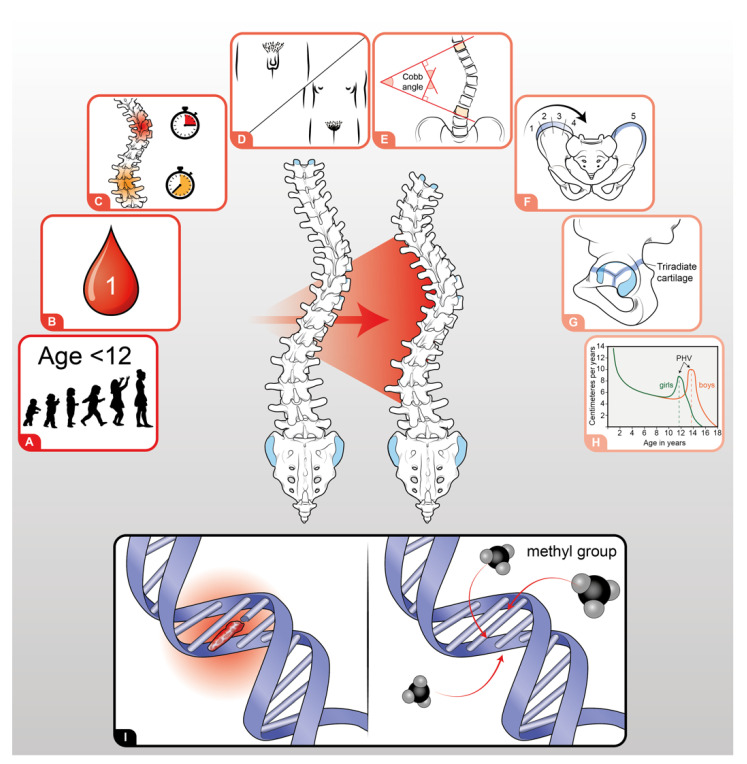
Parameters related to AIS ordered following their impact on scoliosis progression: (**A**) Age < 12 years; (**B**) premenarche status; (**C**) localization of the main curve; (**D**) Tanner stage; (**E**) main curve Cobb angle at diagnosis; (**F**) Risser Stage; (**G**) status of triradiate cartilage; (**H**) high peak velocity; (**I**) genetic (**left**) and epigenetic (**right**) factors.

At the same time, identifying predictors of curve progression is still fundamental to avoid erroneous clinical management depriving patients of adequate treatment or exposing others to unnecessary one. For this purpose, many clinical parameters are widely accepted as predictors of scoliosis progression: curve location, age at diagnosis (<12 years), pre-menarche status, low Tanner stage, and peak height velocity [4,7]. Moreover, some radiographic parameters are currently considered by clinicians, such as curve magnitude at the time of diagnosis (>25°), Risser stage (0–1), open triradiate cartilage, and demonstration of significant curve progression between serial radiographs [6,7]. Figure 1 represents the parameters related to scoliosis progression.

Epidemiological and genetic studies indicated AIS as a polygenic disease, and several studies investigated genetic and epigenetic factors associated with an increased risk of the onset of the scoliotic curve [8,9,10,11]. Several loci associated with AIS susceptibility were identified and evaluated in different ethnic groups, even if the value of AIS susceptibility in clinical practice is limited. Less information is available regarding candidate genetic and epigenetic factors related to scoliotic curve progression and its prediction, which would be a key tool for disease management.

Considering the significant socio-economic burden and psychological effects of a long-term follow-up and risk–benefit ratio of medical intervention, and that clinical features appear inadequate to predict disease evolution, the identification of reliable genetic factors associated with progression could be of crucial relevance in the clinical practice. Genetic and epigenetic markers are potential prognostic tools to predict progression and therefore helpful for personalized treatments and disease management.

The aim of this study is to review the available literature regarding the epigenetic and genetic factors that are associated with the risk of curve progression in patients with adolescent idiopathic scoliosis, to help clinicians in identifying those who can benefit from treatment and a long-term follow-up in this subset of patients.

## 2. Materials and Methods

### 2.1. Review Design

A review of the literature was carried out following the Preferential Reporting Items for Systematic Reviews and Meta-Analyses (PRISMA) guidelines [12].

The Oxford level of evidence scale [13] was used to assess the level of evidence of the included studies. The full version was used to assess randomized and non-randomized clinical trials, whereas the modified version was used to assess all other studies.

Inclusion criteria considered papers describing genetic and epigenetic factors associated with AIS curve progression published in English peer-reviewed journals. Isolated case reports/series with less than 5 patients, literature reviews, and meta-analyses were excluded. The included articles met the PICO criteria for systematic reviews (Population, Intervention, Comparison, and Outcomes). Different types of studies were considered for inclusion: case series, case-control, cohort studies, comparative studies, genome-wide association studies, and case-only studies. These studies were conducted either retrospectively or prospectively.

### 2.2. Search Strategy

Pubmed-MEDLINE, The Cochrane Central Registry of Controlled Trials, Google Scholar, and the Embase Biomedical Database were searched over the years 1990–2022 to identify eligible studies in the English literature describing the genetic factors associated with AIS curve progression. The online literature search was conducted in January 2022 by three reviewers (MM, FB, and GV). The authors stated the following research question: “*Are there genetic and epigenetic factors correlated with scoliotic curve progression in adolescent idiopathic scoliosis patients?*”. This research question matched all four PICO concepts. Subsequently, the following key concepts were formulated “Adolescent Idiopathic Scoliosis”, “curve progression”, “curve severity” and “genetic variants”, “epigenetic variants”, and “polymorphism”, and various alternative terms were considered for each key concept to include the maximum number of articles available in the literature pertaining to the research question. Details on the search strategy are summarized in Supplementary Table S1.

The following search items were combined to perform the search: ‘adolescent idiopathic scoliosis’, ‘gene’, ‘curve progression’, ‘disease progression’, ‘polymorphism’, ‘epigenetic’, and ‘evolution’. 

### 2.3. Study Selection

**Figure 2:**
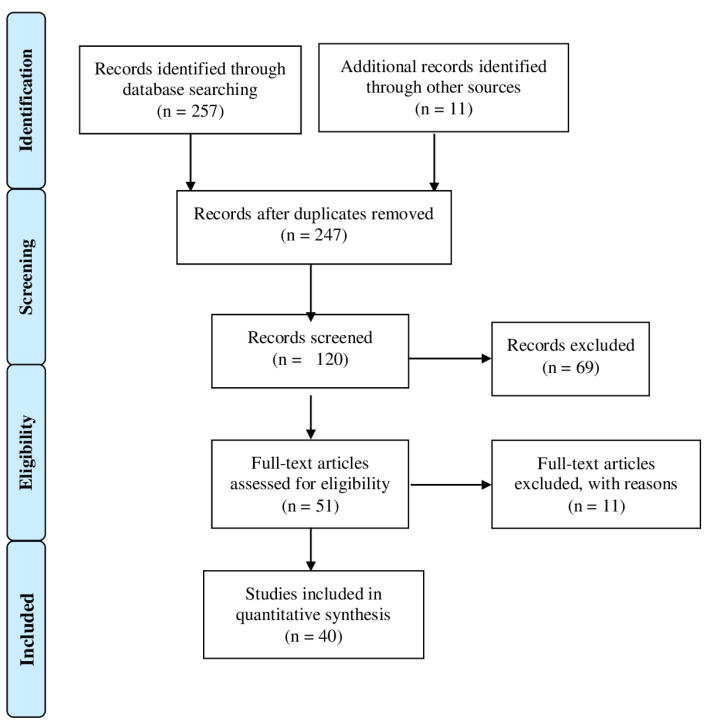
Prisma 2009 flow diagram of the included studies.

After screening the titles and abstracts, the full-text articles were obtained and reviewed. A manual search of the bibliography of each of the relevant articles was also performed to identify potentially missed eligible papers. Duplicates were removed. The study selection process carried out in accordance with the PRISMA flowchart is shown in Figure 2. The present systematic review was accepted for registration in the PROSPERO database for systematic reviews [14] (ID: CRD42022322089).

### 2.4. Data Extraction

Two reviewers (MM and SN) extracted the data through a standardized data collection form. Three reviewers (MM, SN, and AR) checked the data for accuracy, and inconsistent results were analyzed for discussion. The extracted data concerning the study design (with the level of evidence), number of patients, demographics of patients, curve progression definition, biological sample, gene/s involved, mutation/s, and results are summarized in Table 1. The following outcomes were considered for analysis: curve severity defined as the Cobb angle; curve progression measured as the increase in the Cobb angle from the initial evaluation; epigenetic or genetic factors associated with curve progression; and clinical features of curve progression: curve location, age at diagnosis (<12 years), pre-menarche status, low Tanner stage, and peak height velocity time. Moreover, we considered some radiographic parameters currently considered by clinicians, such as the curve magnitude at the time of diagnosis (>25°), Risser stage (0–1), and open triradiate cartilage.

### 2.5. Methodological Quality Assessment of Included Studies

The assessment of the methodological quality of the studies was performed using checklist criteria. The quality assessment tool adopted from the National Institutes of Health/National Heart, Lung, and Blood Institute was used [15]. After answering a series of multiple-choice questions, the quality of each study was reported as poor, fair, or good. All details are summarized in Supplementary Table S2.

## 3. Results

### 3.1. Included Studies

According to the research performed, a total of 40 papers [16,17,18,19,20,21,22,23,24,25,26,27,28,29,30,31,32,33,34,35,36,37,38,39,40,41,42,43,44,45,46,47,48,49,50,51,52,53,54,55] met the inclusion criteria and were considered for review. Of these studies, twenty-one [18,19,21,24,31,33,35,36,37,38,39,40,41,42,44,45,47,50,54,55] were retrospective case-control studies, eight [16,17,27,28,46,48,49,53] were retrospective case series, and six [23,25,26,43,51,52] were retrospective cohort studies. In addition, there was one [30] Genome-Wide Association Study (GWAS), one [34] prospective case-control study, two [29,32] case-only studies, and one [22] retrospective comparative study.

According to the Oxford level of evidence scale, ten [16,17,27,28,29,32,46,48,49,53] of the included studies had a level of evidence IV, twenty-six [18,19,20,21,23,24,26,31,33,34,35,36,37,38,39,40,41,42,44,45,47,50,51,52,54,55] studies had a level of evidence III, while the remaining studies had a level of evidence II [22,25,30,43].

The studies analyzed both small and large-sized populations (*n* = 16 to 2645), describing the association between genetic and epigenetic factors involved in AIS curve progression.

The included studies are heterogeneous (or lacking data) in ethnicity, spine deformity, gender, and curve progression definition (Table 1).

### 3.2. Cohort Characteristics

The studies included in the search reported data on a total of 22,223 patients who underwent genome sampling and analysis, including 16,094 females (72.4%) and 1021 males (4.6%). The median age at the first visit ranged from 12.2 ± 1.2 to 18.5 ± 1.8 years and the median follow-up ranged from 12 months to 42 months. Asiatic populations (Chinese, Japanese, and Korean) [9,16,17,20,21,23,28,29,30,31,32,33,34,35,36,37,38,39,40,42,43,44,45,47,51,53,54,55,56] were the most studied by authors, but Caucasian populations (Europeans, Polish, and French-Canadian) [24,25,26,27,41,43,46,48,49,52], Russian [19], and Turkish [18] populations were also evaluated for possible associations. Two studies did not accurately describe the population demographics [30,43].

### 3.3. Spine Deformity Evaluation 

A detail of the type of scoliotic curve was reported in eleven [16,17,19,22,24,39,40,42,47,51,53] of the selected studies (Table 1), for a total of 3949 thoracic curves (56.8%), 1019 lumbar curves (14.6%), 482 thoraco-lumbar curves (6.9%), 1304 double curves (18.7%), and 195 triple curves (2.8%).

In other studies [23,33,34,37,38,41,49,54,55], the diagnosis was generic or without accurate subtype distribution (i.e., thoracic, or thoracolumbar curve) or not reported [18,20,21,25,26,27,28,29,30,31,32,35,36,43,44,45,46,48,52,56].

As for the initial Cobb Angle, it was accurately described in twenty-three studies [16,17,18,19,21,26,27,29,31,32,34,35,37,39,40,41,44,45,46,47,48,49,50,51,55] with a median angle at first visit ranging from 20.1° ± 8.3° to 77.4° ± 16.1°; other studies reported the range of values or the minimum/maximum values [20,22,23,25,28,33,36,38,42,51,52,53,54].

Regarding the definition of curve progression, the included studies reported the following criteria: increase in the Cobb angle of more than 5° from initial evaluation [16,17,34,39,53,54] or more than 12° every year [41] or any increase on two consecutive X-ray exams taken six months apart [24], Cobb angle exceeding 30°, 40°, 45°, or 50° in an individual not growing [22,25,27,29,30,32,33,42,47,51,52], and a combination of different criteria including an increase in the Cobb angle and/or surgical correction and/or reaching skeletal maturity or not [22,25,52,53]. Twenty-four studies [18,19,20,21,23,26,28,30,31,32,35,36,37,38,40,43,44,45,48,49,50,55] did not specify criteria for spine deformity progression. 

### 3.4. Genetic Factors Associated with Disease Progression

Genetic factors possibly influencing the progression of adolescent idiopathic scoliotic curves were analyzed on genomic DNA prevalently obtained from peripheral blood, or alternatively, from saliva [25,27,43,52].

Numerous polymorphisms were described as associated at different levels with scoliosis curve progression (Table 1), and related genes were hypothesized for their possible involvement in disease development.

Various genes with statistically significant evidence with AIS curve progression were described: Estrogen receptor alfa and beta (*ER*) [16,17,24,39,51,53], Insulin-like growth factor 1 (*IGF-1*) [21,28], Matrillin 1 gene (*MATN1*) [50], Calmodulin 1 gene (*CALM 1*) [51], Tryptophan hydroxylase 1 (*TPH-1*) [53], Neurothropin 3 (*NFT3*) [54], Interleukin 17 receptor (*IL-17RC*) [55], Ladybird homebox 1 (*LBX1*) [20], Lysosomal-associated transmembrane protein 4 beta (*LAPTM4B*) [21], Basonuclin 2 (*BNC2*) [31], Fibrillin 1 or 2 (*FBN1/2*) [23,37], Tissue inhibitor of metalloproteinase 2 (*TIMP 2*) [41], SRY-box transcription factor 9 (*SOX9*) [43], chromodomain helicase DNA binding protein 7 (*CDH7*) [46], Transforming growth factor beta 1 (*TGF-B1*) [19], and microRNA 4300 (*MIR4300*) [47]. Five retrospective studies [25,26,27,29,52] analyzed the association of multiple indices developed by combining 53 different gene SNPs and the initial Cobb angle (“ScoliScore” test) with non-progressive or progressive AIS. Of these, three [10,27,52] showed a significant association of the Scoliscore with different grades of curve progression. Only *ER*-, *IGF-1*-, *FBN1*-, and *MIR4300*-associated polymorphisms and the “Scoliscore” SNPs were successfully replicated in different populations [16,17,21,22,23,37,51]. 

In more detail, Ward et al. [52] investigated the predictive value of the Scoliscore in Caucasian AIS patients, suggesting that a risk model of patients’ natural history could be possible by extracting SNPs from patients’ DNA. The prognostic score, ranging from 1 to 200, was applied to three different cohorts with known AIS outcomes (low-risk females, high-risk females, and high-risk males, where high scores corresponded to a higher risk of curve progression and vice versa). Indeed, low-risk scores (<41) had a negative predictive value close to 100% for each of the three cohorts studied.

The promising “Scoliscore” results were not entirely replicated in the Chinese population by Xu et al. [29], with only two SNPs (rs9945359 and rs17044552) found to be associated with curve progression and severity [29]. The authors stated that, despite the existing ethnic differences (Caucasian vs. Chinese), AIS patients could share two SNPs as common traits in the pathogenesis of curve progression, but the Scoliscore was not reliable in the Chinese Han population.

Similar results were obtained by three other independent studies [25,26,27] analyzing the validity of the Scoliscore in Caucasian [25,27] and French-Canadian populations [26]. 

Putting all these findings together, it may be hypothesized that ethnic differences between Asian and Caucasian populations could yield great divergence regarding the prognostic power of “Scoliscore”. Moreover, the result was not replicated in studies with the same Caucasian population.

Insulin-like growth factor 1 (*IGF-1*) has an important role in skeletal growth [57], representing a good candidate to play a role in AIS curve progression. Yeung et al. [28] first reported a weak association (*p* = 0.04) between the *IGF-1* polymorphism and a higher Cobb angle in Chinese AIS patients, suggesting *IGF-1* as a disease-modifying gene rather than an AIS-onset gene per se.

This result was not replicated in the Japanese population [58], but an association (*p* = 0.01) was described between the rs5742612 polymorphism in the upstream region of the *IGF-1* gene and disease risk, with a significantly different distribution of *IGF-1* genotypes in low- and high-risk groups in the Korean population [21].

The estrogen receptor (*ER*) gene has been shown to be expressed in both human osteoclasts and osteoblasts and plays a critical role in cellular proliferation in bone tissue [59]. Based on the assumption that the estrogen reaction to skeletal and sexual growth is genetically determined by *ER* gene polymorphism, Inoue et al. [16,17] and Zhao et al. [51] found *ER1* gene polymorphism (Xbal site) to be related to curve progression. However, in Tang et al.’s [39] study, a subgroup of Chinese skeletally immature patients was followed until skeletal maturity at age 16, and the abovementioned hypothesis was not confirmed.

Other successfully replicated genetic factors are *FBN1* and *FBN2* variants. The *FBN1/2* genes encode fibrillin, a glycoprotein of the extracellular matrix, and mutations in these genes have been reported in a variety of fibrillin-related disorders (i.e., Marfan syndrome [60]). 

To determine whether *FBN1* and *FBN2* variants were associated with AIS curve progression, Buchan et al. [23] and Sheng et al. [37] found that rare mutations in *FBN1* and *2* were particularly present in severe AIS cases when compared to non-severe cases or healthy controls.

Most of the previously reported associations between genetic markers and AIS curve progression were not replicated in other independent studies. Therefore, Ogura et al. [30] and Wang et al. [47] explored the functional role of the rs35333564 variant located in the *MIR4300HG* gene in different ethnic populations (Japanese and Chinese). Both studies confirmed that the *MIR4300HG* functional variant could significantly add risk of curve progression with similar odds ratios and *p*-values. Moreover, Wang’s study [47] evaluated the relative expression of *MIR4300* in paraspinal muscles among surgical patients carrying different *MIR4300* genotypes, discovering that the GG genotype showed remarkably lower tissue expression than the AA genotype. Interestingly, and for the first time, the tissue expression level of *MIR4300* was significantly correlated with curve severity. To the authors’ best knowledge, there are no studies that contradict the abovementioned association.

Altogether, available data on genetic factors correlated with AIS evolution do not allow the prediction of disease progression based only on genetic information.

Table 2 summarizes the findings concerning genetic factors associated with AIS progression, statistical significance, and the sensitivity/specificity of each variant.

### 3.5. Epigenetic Factors Associated with Disease Progression

In eukaryotes, gene expression is dynamically regulated at the chromatin level by epigenetics, defined as heritable and reversible changes in gene expression without alterations of the underlying DNA nucleotide sequence [61]. Epigenetic marks principally include DNA methylation (the addition/removal of methyl groups to/from cytosines within CpG dinucleotides) and histone post-translational modifications (such as methylation, acetylation, phosphorylation, ubiquitination, and sumoylation). These modifications give rise to local chromatin remodeling that, in turn, modifies the accessibility of regulatory elements to genes. Regulation by non-coding RNAs such as microRNAs is also part of epigenetics. Epigenetic mechanisms regulate cell differentiation and development and are involved in human disease [62].

To date, few studies concerning epigenetic factors involved in AIS progression have been published, but literature data strongly encourage further research in this field.

Meng et al. [34], for the first time, reported a large-scale genome-wide analysis to establish a prognostic model based on methylation status. They analyzed peripheral blood cell DNA of two monozygotic twin pairs discordant for disease progression and validated the results in additional samples. They found a positive correlation between cg01374129 site demethylation and AIS progression (AUC value of 0.805 in the ROC analysis), suggesting epigenetic regulation. Since this site is near the *HAS2* gene (hyaluronan synthase 2), playing a critical role in vertebral and intervertebral disc development, they speculated cg01374129 hypomethylation deregulates *HAS2* expression, impairing normal spine development and causing scoliosis progression. 

Another study [48] used a genome-wide methylation approach to test the influence of DNA methylation status on curve severity, by studying DNA from peripheral blood cells of eight monozygotic twin pairs. The authors found four probes (cg02477677, cg12922161, cg16382077, and cg08826461) where increasing curve severity was associated with hypomethylation. Candidate genes affected by differential methylation include the *WNT* signaling pathway and neuropeptide Y.

Mao et al. [36] investigated promoter methylation of the *COMP* gene, encoding the cartilage oligomeric matrix protein as a target gene for AIS curve progression. *COMP* promoter methylation, associated with low gene expression, was found to directly correlate with AIS curve severity (high Cobb angle of the main curve).

*PITX1* (pituitary homeobox 1, a member of the RIEG/PITX homeobox transcription factors) gene promoter hypermethylation in peripheral blood cells of AIS patients is significantly associated with the Cobb angle of the main curve, suggesting a relationship with disease progression [38]. Similarly, average protochaderin 10 (*PCDH10*) promoter methylation was higher and gene expression was lower in AIS patients compared to controls. Moreover, high *PCDH10* promoter methylation was associated with the Cobb angle of major curves in AIS patients [44]. Furthermore, in this case, data were obtained by analysis of DNA from peripheral blood cells.

In paravertebral muscles, *H19* and *ADIPOQ* genes have been shown to be expressed inconsistently [40], with lower H19 levels and higher ADIPOQ levels in concave-sided muscle tissues compared to convex-sided ones. These data positively correlated with the spinal curve and age at initiation [40], suggesting an important role of H19 and ADIPOQ not only in the onset but also in the progression of AIS. 

On the contrary, the methylation status of estrogen receptor 2 (*ESR2*) in deep paravertebral muscles was found to be associated with the occurrence but not progression of AIS [63].

In another study, the methylation status of tissue-dependent and differentially methylated regions (T-DMRs) of the *ESR1* estrogen receptor was analyzed in superficial and deep paraspinal muscles to explore the association with AIS progression. The authors found suggestive evidence that methylation status might be associated with disease severity [49].

MicroRNAs are small noncoding RNAs that also participate in the regulation of bone metabolism, osteoclast, and osteoblast function. These molecules are epigenetic factors involved in the control of specific molecular pathways in bone-related disorders. 

By performing miRNA expression profile analysis on plasma samples from severe and mild AIS patients and controls, Wang et al. [45] suggested *miR-151a-3p* as a putative biomarker of severe AIS since it was overexpressed in severe but not mild AIS patients. *MiR-151a-3p* may contribute to scoliosis progression through the inhibition of *GREM1* gene expression in osteoblasts interrupting bone homeostasis.

Via microarray analysis, miRNA-145-5p (*miR-145*) and β-catenin mRNA (*CTNNB1*) were found to be overexpressed in AIS bone tissue and primary osteoblasts compared to controls. Significant negative correlations between circulating miR-145 and serum sclerostin, osteopontin, and osteoprotegerin were noted in patients with AIS. The observed aberrant miRNA expression inhibited osteocyte function via Wnt/β-catenin signaling, appearing dysregulated in AIS. *MiR-145* was therefore suggested as a prognostic AIS biomarker [35].

In summary, the hypomethylation of some DNA regions, the hypermethylation of some gene promoters (*COMP*, *PITX1*, *PDCH10*), and the overexpression of some miRNAs (*miR-145*, *miR-151a-3p*) were associated with AIS progression.

Table 3 summarizes the available data on epigenetic factors associated with AIS progression, the techniques used, the tissues analyzed, and the statistical evidence.

## 4. Discussion

Adolescent idiopathic scoliosis (AIS) is the most common type of scoliosis, a complex phenotype resulting from the interaction of multiple genetic loci with each other and the environment [53]. 

AIS is a progressive musculoskeletal disease that may result in cosmetic deformity, back pain and functional deficits, psychological problems, and impaired social interactions [64,65]. Among patients initially diagnosed with AIS, curve progression before skeletal maturity occurs in approximately two-thirds of cases, and in 10% of patients, it progresses to severe scoliosis (Cobb angle >40°) in the following years [6,66]. Although X-ray exams and clinical examinations are currently considered the gold standard for AIS follow-up, they have limited sensitivity and specificity values and provide limited information on curve progression risk [5]. Serial radiographs can result in relatively high cumulative radiation doses, leading to stochastic effects with long-term increased cancer and mortality risks [67]. A recent AIS cohort study stated an overall cancer rate (mostly breast and endometrial) that was five times higher in AIS patients followed up with X-ray exams than the general population [68]. Surgical intervention is currently the ultimate solution established for patients with a severe curve or with conservative treatment failure [69]. It can achieve powerful curve correction but is characterized by high morbidity and intra and/or post-operative complications [70,71].

The control of curve progression is therefore a crucial clinical task, but its etiology is still largely unknown; therefore, new biomarkers are needed to facilitate early detection and accurate curve progression risk assessment. The identification of such biomarkers has the potential to improve patient management, minimize unnecessary orthopedic intervention, define the best applicative protocol for orthopedic treatment, and identify the subpopulation of patients in which early surgery, even with non-severe curves, can avoid operating on severe curves with worse outcomes and more risks. Since clinical features do not adequately predict disease progression, more reliable prognostic factors need to be identified to increase the accuracy of the predictive model, and genetic/epigenetic markers might represent ideal candidates for AIS management. Although the role of genetic factors in AIS development is widely accepted, their role in disease progression is still under study.

In the present work, we systematically reviewed the available literature from 1990 to the present date, concerning genetic and epigenetic factors associated with AIS progression.

Forty papers met the inclusion criteria of the present review, with fifteen genes reported as having SNPs with a significant association with progressive AIS [25,26,27,29,52]. We also considered the development of a predictive algorithm based on a panel of 53 SNPs associated with AIS curve progression, the so-called “Scoliscore”, whose ability to discriminate between patients with a low or high risk of progression failed to be replicated in some populations [25,26,27,29,52].

Available data concerning genetic factors suggest a relatively low association and, if present, an association with low predictive capacity (Table 1 and Table 2), low odd risk values, and low level of evidence (III or IV). Moreover, the low replicability in different ethnicities confirms the extreme variability of the genetic influence on curve progression, suggesting its multifactorial nature, as is the case for AIS onset. Of the 15 genes reported as having SNPs with a significant association with progressive AIS, none showed sufficient power to sustain clinical applications.

Discordant AIS progression described in monozygotic twins [37] suggested the involvement of nongenetic factors and epigenetic processes are emerging as the best candidates [37], with a series of genes whose methylation was correlated with AIS curve severity [34,36,38]. Nine studies reporting epigenetic modifications showed promising results in terms of reliable markers suggesting epigenetics as the more promising field for the identification of factors associated with AIS progression, offering a rationale for further investigation in this field.

To the best of our knowledge, this is the first systematic scoping review where the available evidence evaluating the genetic and epigenetic factors influencing AIS curve progression was analyzed and, if necessary, integrated with additional calculations. Moreover, this work included an analysis of epigenetic factors, focusing not only on hereditable factors but also on the importance of environmental influences and tissue-related genetic expression on the AIS phenotype.

The main limitation of the present review is the presence of high heterogeneity among the included studies in terms of a lack of homogeneous study design and prospective comparative studies with high values of associations and predictive capacity, possibly representing the principal selection bias of the present work. Moreover, the absence of a clear, internationally recognized definition of progression of the curve and the low replicability of association between SNP and AIS progression in different populations generate non-reliably comparable conclusions and represent a confounding factor. The number of published papers on genetic and epigenetic factors related to AIS progression is noteworthy and surprising but without a final international consensus. Defining the factors related to AIS curve progression has the potential to completely renew the clinical management of such a frequent disease.

On the other hand, as more AIS progression-associated variants are identified, they could be incorporated into a “risk of progression scoring system” that can predict the risk of progression. Artificial intelligence may be used for this purpose, thanks to the development of algorithms based on deep learning and machine learning, employing data from spine radiographs, clinical patients’ features, and genetic/epigenetic factors to create a complete “tailored” diagnostic tool. Although this approach is fascinating, no clinical studies have attempted this approach.

Therefore, in the forthcoming years, different new biomarkers could be combined with clinical and radiographic parameters, hopefully for the development of new therapeutic strategies based on genetic factors and epigenetic modulators. In line with this mission, further prospective comparative studies with homogeneous architecture and cohorts are needed.

## 5. Conclusions

In conclusion, prognostic testing for AIS has the potential to significantly modify disease management. This will be achieved only after the identification of reliable markers and an understanding of the underlying biologic pathways. Genetic studies identified a series of loci associated with disease progression, whose power appears, however, insufficient to guide clinical choices. More recent evidence suggests epigenetics as a more promising field for the identification of factors associated with AIS progression, offering a rationale for further investigation in this field. More data are needed, and studies on tissues involved in the pathology, rather than peripheral blood, are necessary.

## Figures and Tables

**Table 1:** Details of the included studies. (NS = non specified).

Study Design (Level of Evidence)	Study Population	Age (Mean/Range) Gender	Ethnicity	Spine Deformity	Initial Cobb Angles (Mean/Max)	Follow-Up Period	Curve Progression Definition	Biologic Sample	Technique	Gene/s Involved Polymorphism	Results	Authors
Retrospective case series (IV)	304 girls with AIS (main curve < 10°)	12.5 ± 1.6 years 100% female	Japanese	189 double curves 62 right thoracic curve, 25 thoracolumbar curves 13 lumbar curves 15 triple curves	24.6 ± 10.0° 31.3 ± 12.6°	>1 year until growth maturation when height no longer changes	progression of 5° from initial evaluation	DNA from peripheral blood lymphocytes	PCR-RFLPs	ER gene Polymorphic first intron PvuII site and the XbaI site	XbaJ polymorphism in the ER gene associated with curve progression (*p* = 0.03).	**M. Inoue (2002)**
Retrospective case series (IV)	304 girls with AIS (main curve < 10°)	12.5 ± 1.6 years 100% female	Japanese	189 double curves 62 right thoracic curve, 25 thoracolumbar curves 13 lumbar curves 15 triple curves	24.6 ± 10.0° 31.3 ± 12.6°	>1 year until growth maturation when height no longer changes	progression of 5° from initial evaluation	DNA from peripheral blood lymphocytes	PCR-RFLPs	MED4 ESR1 CYP17A1	The XbaJ polymorphism in the ER gene was associated with curve progression (*p* = 0.03).	**M. Inoue** **(2002)**
Retrospective case series (IV)	340 AIS female patients	12–16 100% female	Chinese	NS	≥20°	Until skeletal maturity, 16 years, or surgical intervention	NS	Peripheral blood sample	PCR-RFLD	IGF-I Rs5742612 and rs2288377	Cobb’s angle higher in patients with TT genotype *p* = 0.04)	**Y. Yeung** **(2006)**
Retrospective Case-control study (III)	540 AIS patients 260 healthy controls (A subgroup of 364 AIS patients had been followed up to skeletal maturity at age 16)	AIS patients: 13.4 ± 1.4 Female: 540 (100%) Healthy controls: 13.3 ± 1.3 Female: 260 (100%)	Chinese	-King III (24.9%) -thoracolumbar (22.6%). -King I 14.5% -King II 16.2% -King V (6.8%) -lumbar curve (8.3%) -triple curve (6.6%).	28.9° ± 11.5°	Until skeletal maturity at age 16	Curve progression was defined as increase in Cobb angle with greater than 5° from the initial evaluation.	Peripheral blood sample	PCR-RFLD	ER gene Two common SNPs (PvuII and XbaI) in the intron 1 of ESR1	No association between curve severity and curve progression and the two SNPS (Pvull and XbaI)	**N. Tang** **(2006)**
Retrospective Case-Control Study (III)	419 AIS patients 750 healthy controls	AIS patients: 16.1 ± 0.93, (12–19), 89.8% female Healthy controls: 15.8 ± 0.10, (8–24), 88.3% female	Chinese	NS	High-risk genotype: 32.11° ± 11.67° Intermediate-risk genotype: 32.25° ± 12.42° High-risk genotype: 37.91° ± 17.1°	more than 12 months until the age of growth maturation (16 years old)	NS	Peripheral blood leukocytes	PCR-RFLD	MATN1 gene (matrilin 1 gene): rs1188402, rs1065755 rs1149045, rs1149046, rs3828051, rs1149048, rs12404006	Genotype GG of Rs1149048 SNPs was statistically significant with the mean maximal Cobb angle.	**Z. Chen** **(2009)**
Retrospective cohort study (III)	67 AIS patients with double curve 100 healthy controls	AIS patients: 15.09 ± 2.37, 10–20 7.8% male 82.2% female Healthy controls: 15.55 ± 2.21, 10–19 25% male 75% female	Chinese	40 thoracic curves 12 thoracolumbar curves 15 lumbar curves	The Cobb angle of the major curve of AIS ranged from 30° to 90°. There were 60 patients with Cobb angle >40°.	NS	Cobb angle >30	Peripheral blood sample	PCR	ER1 CALM1 ER1 gene: rs2234693, allele T; CALM 1 gene: rs12885713, allele T	Significant association between double curve and CALM1 ER1 SNPs, and between Cobb angle and SNPs of ER1 gene (0.0128)	**D. Zhao** **(2009)**
Retrospective Cohort study (III)	Screening group (277): -Severe: 8 (3%) -Moderate: 34 (12%) -Mild: 235 (85%) Spine surgery practice group (257): -Severe: 28 (11%) -Moderate: 54 (21%) -Mild: 175 (68%) Male group (163): -Severe: 18 (11%) -Moderate: 18 (11%) -Mild: 127 (78%)	9–13 at diagnosis Screening group: Female: 277 (100%) Spine surgery group: Female: 257 (100%)	Caucasian	NS	>10°	Until skeletal maturity or sever curve	-Progression to a severe curve: Cobb angle >40° in an individual still growing. Cobb angle >50° in an individual not growing -Progression to a moderate curve: Cobb angle of 25° or greater but not reaching the severe range by skeletal maturity	Saliva samples	Quantitative PCR	53 SNPs identified with a previous GWAS The authors stated a prognostic test algorithm (AIS-PT, Scoliscore) with a scale (1–200) based on 53 SNP markers, cut point 40: 1–40 (≤1% risk of progression)	Low-risk scores (<41) had NPV of 100%, 99%, and 97%, respectively, in the tested populations. (95% CI: 98.6–100.0).	**K. Ward** **(2010)**
Retrospective case series (IV)	312 AIS patients: -90 failures of the brace treatment -222 successes of the brace treatment	12.7 ± 1.5, (10–15) Female: 41 (87%) Male: 41 (13%)	Chinese	Single thoracic curve 128 (32.1%) Single thoracolumbar or lumbar curve 66 (30.3%) Double major curve 118 (24.5%)	<30: 195 patients ≥30: 117	14.4 ± 4.8 months, (7.2 ± 26.4)	Curve progression of more than 5° compared to the initial Cobb angle Surgical intervention because of curve progression.	Peripheral blood sample	PCR-FLP	Single nucleotide polymorphism (SNP) sites in the genes for estrogen receptor a (rs9340799), estrogen receptor b (rs1256120), tryptophan hydroxylase 1 (rs10488682) melatonin receptor 1B (rs4753426), and matrillin-1 (rs1149048),	Statistically significant differences between the two groups in SNP rs9340799 in ERa.	**L. Xu** **(2011)**
Retrospective Case-control study (III)	362 AIS patients 377 age-matched controls 120 skeletally immature AIS patients who received continuous brace treatment for minimum of 2 years	AIS patients: 15.30 ± 2.49 10–20 91.9% female Controls: 15.86 ± 0.93 14–18 90.2% female	Chinese	Thoracic and thoracolumbar curves	25°–40°	30 ± 4.2 months	Curve progression of more than 5° compared to the initial Cobb angle	Peripheral blood sample	PCR-RFLP	NTF3 gene: rs1805149 SNP rs11063714 SNP	rs11063714 SNP significantly associated with lower mean maximum Cobb angle and brace treatment success	**Y. Qiu** **(2012)**
Retrospective case-control study (III)	529 AIS case 512 healthy controls	AIS case: 14.54 ± 1.62 (1–18) Female: 529 (100%) Healthy controls: 14.36 ± 1.93 (11–18) Female: 512 (100%)	Chinese	Thoracic curve	AIS case: Mean Maximum Cobb: 38.30° ± 16.71°, (20°–100°)	NS	NS	Peripheral blood sample	PCR-RFLP	IL-17RC gene Rs708567	GG genotype of Rs708567 showed significant association with higher Cobb angle	**S. Zhou** **(2012)**
Retrospective Case-Control study (III)	53 cases of AIS 54 controls	AIS group: 14.9 ± 3.4 9–19 Females: 46 (86.8%) Males: 7 (13.2%) Control group: 29.8 ± 5.5 18–40 Females: 51 (94.4%) Males: 3 (5.6%)	Turkish	NS	29.88° ± 11.78°	NS	NS	Peripheral blood samples	RT-PCR	MCM6: 6p21; 13910; 4988235 MATN1: 1p35; 1149048 VDR BsmI: 12q13.1; 1544410	There was no statistical difference (*p* < 0.05) between case and control in terms of progression of the curve	**H. Yilmaz** **(2012)**
Retrospective case-control study (III)	300 AIS patients 300 Healthy controls	AIS group: -12.8 ± 2.1 Female 156 (52%) Male: 144 (48%) Controls: 13.3 ± 2.8 Female: 160 (53.3%) Male: 140 (46.7%)	Russian	Thoracic: 167 (56.1%) Thoraco-lumbar: 117 (39.2%) Lumbar: 14 (4.7%)	10°–19°: 154 (51.3%) 20°–29°: 116 (38.7%) 30–39°: 20 (6.7%) <39°: 10 (3.3%)	36 months	NS	Peripheral blood sample	RT-PCR	TGF! Rs1800469 Rs1800471	TGFB1 gene is associated with curve severity and progression in AIS.	**I. Ryzhkov (2013)**
Retrospective Case-control study (III)	949 AIS patients 976 age-matched normal control subjects	AIS group: 820 girls and 129 boys Control group: 662 females and 314 males	Chinese	NS	>20°	NS	NS	Peripheral blood sample	PCR-RFLP	LBX1 (ladybird homeobox 1) gene on chromosome 10q24.31. SNP rs11190870 near LBX1	TT genotype of rs11190870 Significantly associated with larger Cobb angle	**H. Jiang** **(2013)**
Retrospective Case-Control study (III)	68 AIS patients: -33 lower-risk group: Cobb’s angle 10–40 -35 high-risk group: Cobb’s angle >40° 35 age-/sex-matched controls	AIS group: -low-risk: 14.5 26 (80.6%) females 7 (19.4%) males -high risk: 14.9 9 (25%) males 26 (75%) females Control group: 13.4 9 (25%) males 26 (75%) females	Korean	NS	Low risk: 25.8° High risk: 58.8°	NS	NS	Peripheral blood samples	PCR-RFLP	CHL1 (rs10510181) DSCAM (rs2222973) LAPTM4B (rs2449539) FOXB1 (rs1437480) CBLN4 (rs448013) RRAGC (rs10493083) BRIP1 (rs16945692) MATN1 (rs1149048) MTNR1B (rs4753426) IGF1 (rs5742612)	LAPTM4B rs2449539 significantly associated with higher risk of progression.	**E. Moon** **(2013)**
Retrospective Comparative study (II)	2217 AIS patient -progression group (880 patients) -non-progression group (492)	Progression group: 17.2 Female: 830 (94.3%) Male: 50 (5.7%) Non-progression group: 16.8 Female: 469 (95.3%) Male: 23 (4.7%)	Japanese	Thoracic curve: 819 (93%) Non-Thoracic curve: 61 (7%)	>10°	NS	>40° progression group <30° and skeletal maturation Non-progression group	Peripheral blood sample	PCR	neurotrophin 3 (rs1063714) G protein-coupled estrogen receptor (rs3808351, rs10269151 rs4266553) tissue inhibitor of metalloproteinase (rs8179090)	No statistical difference was found between the 4 SNPs and AIS curve progression	**Y. Ogura** **(2013)**
Retrospective cohort study (III)	405 European AIS patients: -rare variants: 26 -No rare variants: 379 370 Chinese Han AIS patients: -rare variants: 28 -No rare variants: 342 47 Other ancestries AIS patients	European AIS -No rare variants: Female: 326 (86%) Male: 53 -Rare variants: Female: 22 (83%) Male: 3 (17%)	European and Chinese	Right thoracic and thoracolumbar curves	>10°	NS	NS	Peripheral blood sample	Exome sequencing	Rare damaging variants of FNB1 and FNB2	FBN1 or FBN2 variant was associated with curve magnitude	**J. Buchan** **(2014)**
Retrospective Case-control study (III)	248 AIS patients: -Non-progressive IS: 90 -Slowly progressive IS: 90 -rapidly progressive IS (RP-IS): 62 243 healthy female controls	NS	Polish	Thoracic curve: 191 (77%) Lumbar curve: 51 (20.5%) Single curve: 97 (39%) Double curve: 145 (58.5%)	NS	3 years	The change of Cobb angle value on 2 consecutive radiographs taken at 6 months of distance	Peripheral blood samples	PCR-RFLP	ESR2 gene: Promoters: Alw NI (rs1256120) AluI (rs4986938) Rsa I (rs1256049)	There was a difference of genotype distribution of rs4986938 between progression and non-progression groups.	**T. Kotwicki** **(2014)**
Retrospective cohort study (II)	126 AIS patients -Progression group: 27 (21%) patients -Non-progression group: 99 (79%)	12.2 ± 1.2 (9–15.8) 113 female (89.7%) 31 males (10.3%)	Caucasian	NS	10°–25°	28.5 ± 9.9 months	Patients who had curve progression to >40 or had undergone a spinal fusion	Saliva sample	Quantitative PCR	Prognostic test algorithm (AIS-PT, Scoliscore) with a scale (1–200). Cut point: Low risk (1 to 50 points), intermediate risk (51 to 179 points), or high risk (180 to 200 points).	No significant association between the continuous ScoliScore value and curve progression (*p* = 0.720).	**B. Roye** **(2015)**
Retrospective cohort study (III)	148 patients with severe AIS: -302 patients with non-severe AIS -901 healthy controls	Severe AIS: 15 ± 2 years (10–25) Females: 129 (87.2%) Males: 19 (12.8%) Non-severe AIS 16 ± 1 years (14–22) Females: 259 (85.7%) Males: 43 (14.3%)	French-Canadian	NS	56° ± 12° (37°–90°)	NS	NS	Peripheral blood sample	Quantitative PCR	The authors stated a prognostic test algorithm (AIS-PT, Scoliscore) with a scale (1–200) based on 53 SNP markers.	None of the SNPs used were associated.	**Q. Tang** **(2015)**
Retrospective case series (IV)	16 AIS patients	12.5 (10–15)	Caucasian	NS	25.2° (20°–33°)	2.3 years (1–4 At least 1 year after brace treatment or skeletal maturity)	Cobb >45°	Saliva sample	Quantitative PCR	Prognostic test algorithm (AIS-PT, Scoliscore) with a scale (1–200). Cut point:160; 160–200 (high risk of curve progression with Cobb >45°) vs. <160 (low risk of curve progression with Cobb >45°)	The mean ScoliScore among those who progressed to more than 45 degrees was higher than that among those who did not (176 vs. 112, *p* = 0.030).	**D. Bohl** **(2016)**
Case-only study (IV)	670 AIS patients -313 in non-progression group -357 in progression group	-Non-progression group: 12.3 ± 2.5 -Progression group: 12.5 ± 2.7 years	Chinese	NS	-22.6° ± 3.7° for non-progression group -53.4° ± 12.7° for progression group.	NS	-Cobb angle <25° at final follow-up: non-progression group. -Cobb angle >40°: progression group.	Peripheral blood sample	Quantitative PCR	The authors stated a prognostic test algorithm (AIS-PT; Scoliscore) with a scale ranging from 1 to 200	Allele A of rs9945359 was significantly higher in the progression group than in the non-progression group (*p* = 0.01).	**L. Xu** **(2016)**
Genome-wide association study (GWAS) (II)	2142 patients with AIS 1105 in progression group 832 in non-progression group 205 patients excluded	NS	Japanese	NS	NS	NS	Progression group: Cobb angle 40° Non-progression: Cobb Angle 30° in skeletally mature patients	Peripheral blood sample	NS	MIR4300 microRNA host gene (SNP rs1828853)	rs1828853 showed association with progression of AIS.	**Y. Ogura** **(2017)**
Retrospective Case-control study (III)	2645 AIS patients 2746 healthy controls (And further replicated in 693 patients and 254 controls.)	12.5 ± 2.1 years for the patients 16.9 ± 2.8 years for the controls	Chinese	NS	56.2 ± 14.3°	NS	NS	Peripheral blood sample and bilateral intraoperative facet joint tissue	SNP Genotyping Assay	BNC2 (rs10738445)	Genotype CC h larger Cobb angle	**L. Xu** **(2017)**
Case only study (IV)	1860 Patients with AIS -594 mild curve -326 moderate curve -940 severe curve	10–18 years Females: 1763 (94.7%) Males: 97 (5.3%)	Japanese	NS	Severe curve: 54.8° ± 12.1° Mild curve: 24.4° ± 4.0°	NS	-Severe curve: Cobb angle of 40) -mild curve: Cobb angle <30.	Peripheral blood sample	PCR-RFLP	LBX1 (ladybird homebox 1) 10q24.31 SNP rs11190870	No significant differences were observed between the groups	**Y. Takashi** **(2018)**
Retrospective case-control study (III)	319 AIS patients 201 age-matched female healthy controls	AIS patients: 14.3 ± 2.2; 10–16 Controls: 13.7 ± 1.2; 10–16	Chinese	major right thoracic curvature and major non-thoracic curvatures	Cobb >10°	Until skeletal maturity or surgery	-Progressive curve group: Cobb >40° -non-progressive curve group: Cobb angle <40	Peripheral blood samples	PCR-RFLP	LBX1, BNC2, SOX9/KCNJ2, GPR126, AJAP4, BCL-2, PAX3/ EPHA4, LBX1 (LBX1- AS1). SNPs: rs11190870, rs12946942, rs13398147, rs241215, rs3904778, rs6570507, and rs678741	There was no association found between the seven SNPs with curve progression in AIS	**G. Man** **(2018)**
Prospective Case-control study (III)	92 AIS patients: -50 patients in the progression group -42 patients in the non-progression group 276 unrelated subjects: -112 in progression group -164 in non-progression group	AIS patients: -Progression group: 13.7 ± 2.4 Female: 37 (74%); Male: 13 (26%) -Non progression group: 13.3 ± 1.9 Female: 30 (71%); Male: 12 (29%) Unrelated subjects: -Progression group: 14.1 ± 2.1 Female: 85 (76%); Male: 27 (24%) -Non progression group: 13.8 ± 2.7 Female: 126 (76.8%) Male: 38 (23.2%)	Chinese	Single thoracic, thoracolumbar, single lumbar, double thoracic, and double lumbar	AIS patients: -Progression group curve > 45° -Non-progression group curve <30° Unrelated subjects: 10° < curve > 25°	Until skeletal maturity	curve progression of at least 5° in two successive clinical follow-ups	Peripheral blood sample	Oligonucleotide Ligation and Detection system	The genome and methylome of peripheral monocytes were sequential	Methylation levels of site Cg01374129 (Has2 gene) were significantly lower in the progression group than in the non-progression group.	**Y. Meng** **(2018)**
Retrospective case-control study (III)	AIS patients: 13 Non-AIS controls: 10	AIS patients: 15.54 ± 1.76 Non-AIS patients: 15.60 ± 5.77	Chinese	NS	AIS patients: 58.15° ± 11.41°	NS	NS	Human bone-derived primary bone cells from iliac crest bone tissue and serum	RT quantitative PCR	MiR-145 of Wnt/ß catenin	Significant negative correlations between circulating miR-145 and serum sclerostin, osteopontin, and osteoprotegerin.	**J. Zhang** **(2018)**
Retrospective case-control study (III)	50 patients with AIS 50 healthy controls	AIS patient: 12.98 ± 1.46 Female: 46 (92%) Male: 4 (8%) Healthy controls: 12.46 ± 1.59 Female: 45 (90%) Male: 5 (10%)	Chinese	NS	29 AIS patients > 40° 21 AIS patients < 40°	NS	NS	Peripheral blood sample	PCR and pyrosequencing	COMP gene promoter methylation	AIS patients with different levels of methylation showed significant differences in Cobb angle of main curve (*p* = 0.011)	**S. Mao** **(2018)**
Retrospective Case-control study (III)	I960 AIS patients 1499 healthy subjects	AIS group: 14.3 ± 3.2 Healthy group: 22.5 ± 5.9	Chinese	All AIS patients had main thoracic curve	AIS patients: 38.58 ± 12.38	NS	NS	Peripheral blood samples Intraoperative muscular tissue	RT-PCR	FBN 1 & FBN 2 106 SNPs of FBN 1 & FBN2	The expression level of FBN1 was remarkably correlated with the curve severity (*p*.0.02).	**F. Sheng** **(2018)**
Retrospective Case-control study (III)	50 patients with AIS 50 healthy controls	AIS patients: 22 patients: 10–13 28 patients: 14–16 Female: 46 (92%) Male: 4 (8%)	Chinese	Thoracic or thoraco-lumbar curve	Cobb from 10° to 50°	NS	NS	Peripheral blood sample	Pyrosequencing	PITX1 PITX1 promoter methylation	The methylation level of 6 CpG sites in PITX1 promoters was significantly associated with Cobb angle.	**B. Shi** **(2018)**
Retrospective Case-control study (III)	5 AIS patients: -10 paraspinal muscle samples 60 Validation non-AIS patients	AIS patients: -14.2 ± 1.92 years -5 female (100%) Validation non-AIS patients: -15.25 ± 2.64 -49 female (81.6%) -11 male (19.4%)	Chinese	AIS patients: -Lenke 1: 3 -Lenke 3: 1 -Lenke 4: 1 Validation non- AIS patients: -Lenke 1: 34 -Lenke 2: 3 -Lenke 3: 15 -Lenke 4: 8	AIS patients: 56.8° ± 6.06° Validation non- AIS patients: 54.48° ± 10.09°	NS	NS	Intraoperative paraspinal muscular samples	RNA sequences + Quantitative RT-PCR	ADIPOQ mRNA and H19 mRNA	ADIPOQ mRNA and H19mRNA showed statistical significance (*p* < 0.001 and *p* = 0.04, respectively)	**H. Jiang** **(2018)**
Retrospective Case-control study (III)	100 AIS patients: -53 progressive curves -47 non-progressive curves 100 healthy controls	AIS patients: 12.7 ± 1.5 Female: 100 (100%) Healthy controls: Female: 100 (100%)	Polish	Right-sided thoracic curve of Cobb angle greater than 20° (Lenke types 1 and 3).	AIS patients: -Whole group: 31.3° -Progression group: 35.4 -Non-progression group: 27.7	34.8 ± 21.6	More than 12° of Cobb angle every year	Peripheral blood sample	PCR-FRET	TIMP2 Nine different TIMP2 polymorphism	Four of the polymorphisms showed non-equal distributions in patients with different progression rates.	**M. Andrusiewicz** **(2019)**
Retrospective Case-control study (III)	223 AIS patients 375 age-matched controls	AIS patients: 127 patients < 12 96 patients > 12 Female:199 (89%) Male: 24 (11%)	Chinese	Lenke 1: 23 Lenke 2: 110 Lenke 3: 28 Lenke 4: 27 Lenke 5: 29 Lenke 6: 6	130 patients < 23° 93 patients > 23°	11.9 (1.4 months to 31 months)	a curve greater than 30° after skeletal maturity was used to define curve progression	Peripheral blood sample	Exome sequencing	The authors searched for rare damaging variants (defined as missense, nonsense, frameshift, or splice-site variants and variants with a minor allele frequency of <1% in public databases)	The number of rare damaging variants associated with curve progression (*p* < 0.05, OR = 4.304, 95%, CI 2.4 to 7.5	**H. Jiang** **(2019)**
Retrospective Cohort study (II)	2272 patients with severe AIS 13,859 healthy controls	NS	Japanese; Chinese and Scandinavian	NS	NS	NS	NS	Peripheral blood or saliva sample	PCR based	17q24.3 near the genes SOX9 and KCNJ2) rs12946942	rs12946942 SNP showed significant association in severe AIS patients:	**K. Takeda** **(2019)**
Retrospective Case-Control study (III)	50 AIS patients 50 Healthy controls	AIS patients: -12.6 ± 1.5 years (10–18 years) Healthy controls: -12.5 ± 1.6 years (10–18 years) 100 females (100%)	Chinese	NS	AIS patients: -20.1° ± 8.3° (10°–60°)	12 months	NS	Peripheral blood samples	RT-PCR	PCDH10 gene methylation and expression	PCDH10 methylation level significantly correlated to curve severity	**B. Shi (2019)**
Retrospective Case-control study (III)	(1) mi-RNA sequencing cohort: 10 AIS patients: -5 severe curves -5 mild curves 5 Healthy controls (2) qPCRT validation cohort: -40 severe curve -40 mild curve -40 healthy controls (3) Facet joints and bone tissues group: -21 severe AIS patients -20 non-scoliosis patients	Mi RNA sequence cohort: -Severe AIS: 13.0 ± 2.5 years 5 female (100%) -Mild AIS: 14.2 ± 0.8 years 5 female (100%) -5 controls: 11.6 ± 2.9 years 5 female (100%) qPCR cohort: -Severe AIS: 12.6 ± 2.2 years 27 females (67.5%) 13 males (32.5%) -Mild AIS: 12.4 ± 1.8 years 30 female (75%) 10 males (25%) -Healthy controls: 12.2 ± 1.9 28 female (70%) 12 male (30%)	Chinese	NS	Mi RNA sequence cohort: -Severe AIS: 64.6° ± 16.7° -Mild AIS: 26.0° ± 5.8° qPCR cohort: -Severe AIS: 61.3° ± 9.5° -Mild AIS: 22.7° ± 6.7°	NS	NS	Peripheral blood sample and bone tissue	RT-PCR	miR-151a-3p and GREM1 expression	miR-151a-3p and GREM1 expression significantly correlated to severe AIS curves	**Y. Wang (2020)**
Retrospective case series (IV)	211 AIS patients: -Non-progressive curve = 80 -Slowly progressive curve = 78 -Rapidly progressive curve = 53 83 healthy subjects	AIS patients: -Non-progressive: 18.5 ± 1.8 (15.0–24.1) -Slowly progressive: 16.9 ± 2.4 (15–26.2) -Rapidly progressive:16.3 ± 7.1 (12.4–50.1) Female: 211 (100%)	Caucasian	NS	AIS patients: -Non-progressive: 23.9 ± 5.4 (10–30) -Slowly progressive: 38.9 ± 7.9 (30–65) -Rapidly progressive: 62.7 ± 15.7 (39–114)	12 months	The change of Cobb angle value on the two consecutive X-rays taken at 12-month time intervals expressed in degrees per month.	Peripheral blood sample	PCR-RFLP for: rs1017861, rs1324842, rs4738813 Sanger sequencing for: rs78874766 rs4738824, rs7479761	CHD7 rs1017861, rs13248429, rs4738813 rs78874766, rs4738824, and rs74797613.	rs1017861 and rs4738813 were associated with curve severity and progression rate (*p* < 0.05).	**K. Borysiak** **(2020)**
Retrospective case-control study (III)	1952 AIS patients: -747 progression group -520 non-progression group 2495 healthy controls	AIS group: -Progression group: 13.2 ± 2.4 -Non progression group: 13.0 ± 2.3 Female: 1952 (100%)	Chinese	-1218 (62.4%) main thoracic curve -476 (24.3%) double major curve -258 (13.3%) major lumbar curves	36.8 ± 3.2, (22–66)	NS	Progression group: -Cobb angle >50° and Risser grade < 3 Non-progression group: -Cobb angle < 30° and Risser grade >3 at final follow-up	Peripheral blood sample 76 intraoperative muscular tissue	RT-PCR	MIR4300 HG gene rs35333564	Significant difference between two groups regarding both genotype frequency and minor allele frequency of rs35333564 in MIR4300 gene.	**Y. Wang** **(2021)**
Retrospective case series (IV)	8 female monozygotic twin pairs (*n* = 16 patients): -6 discordant twin pairs (difference in primary curve Cobb angle > 10°) -2 concordant twin pairs (difference in primary curve < 2°)	All individuals: -37.3 ± 22.5 years Female: 16 (100%)	Caucasian	NS	39.6° ± 15.3°	NS	NS	Peripheral blood sample	Microarray analysis	Genome-wide methylation in blood (Differentially methylation region (DMR) promoter enrichment analyses)	SNPs hypomethylation associated with curve severity	**P. Carry** **(2021)**
Retrospective case series (IV)	29 AIS surgery patients: -10 patients with Cobb ≤70° -19 patients with Cobb >70°	All individuals: 14.5 ± 1.5 years (12.1–17.9) 29 female (100%)	Caucasian	Main thoracic curve	All individuals: 77.4° ± 16.1° (52°–115°)	2 years	NS	Intraoperative deep paraspinal muscles sample and trapezius muscles	PRC and Pyrosequencing	Methylation levels of ESR1 regulatory regions	DRM1/2 methylation status was significantly associated with curve severity	**P. Janusz (2021)**

**Table 2:** The reported SNPs with statistically significant evidence in AIS curve progression (NS = non-specified, OR = odds ratio).

Gene	SNP Risk Allele	Molecular Pathway	Sensitivity/Specificity/OR/CI	*p*-Value	Results	Reference
***ER1* (estrogen receptor 1)**	XbaI site (A/G rs934099)–Genotype Xx	Estrogen determines different skeletal and sexual growth reactions that are genetically determined by the ER gene polymorphism	NS	0.03	The mean (±SD) initial Cobb angle was 27.5 ± 14.8 with genotype XX, 26.2 ± 9.9 with genotype Xx, and 23.3 ± 8.5 with genotype xx, and the differences were statistically significant. XbaJ polymorphism in the ER gene was significantly associated with curve progression.	M. Inoue (2002)
***ER1* (estrogen receptor)**	PvuLL site (rs2234693)	Estrogen determines different skeletal and sexual growth reactions that are genetically determined by the ER gene polymorphism	Sensitivity: 28–69% Specificity: 44–82% Positive predictive value: 45–51% Negative predictive value: 63–68%	0.0128	A significant difference was shown between cases (Cobb angle >40°) and controls in the polymorphic distribution of the rs2234693 (Pvu II) site in the ER 1 gene (P 0.0128). In addition, the frequency of the -16C allele in the cases (73.3%) was less than in the controls (81.5%).	D. Zhao (2009)
***ERalpha* (Estrogen receptor alpha)**	rs9340799-GA and G allele	Estrogen determines different skeletal and sexual growth reactions that are genetically determined by the ER gene polymorphism	Sensitivity: 51% Specificity: 82% OR = 3.559 within 95% Confidence Interval (CI): 0.99–4.38	<0.001	Statistically significant differences between the two groups (progression vs. non-progression) in SNP rs9340799 in ERa (genotype GA (50.9 vs. 17.9) and G allele (27.1 vs. 12.0%). Allele G of ER alpha could be considered as risk factor leading to progression of AIS curve.	L. Xu (2011)
***CALM1* (Calmodulin 1 gene)**	rs12885713	Calmodulin regulates the contractile properties of muscles and platelets through its interaction with actin and myosin and regulates cellular calcium through transport across the cell membrane	Sensitivity: 28–69% Specificity: 44–82% Positive predictive value: 45–51% Negative predictive value 63–68%	0.034	A significant association was found between double curve and polymorphic distributions of CALM 1 SNPs (0.034). A combination of CALM1 and ER1 gene polymorphisms might be related to double curve in patients with AIS, which is associated with curve progression.	D. Zhao (2009)
***IL-17RC* (Interleukin 17 receptor)**	Rs708567-genotype GG	The IL-17R complex mediates the signal transduction of the IL-17 signaling axis. This promotes the production of pro-inflammatory cytokines.	Sensitivity: 94% Specificity: 17% Positive predictive value: 60% Negative predictive value: 69%	0.007	Overall, AIS patients with the GG genotype showed a significantly higher mean maximum Cobb angle (36.01° ± 13.12°, 20°–58°) than those with the AG genotype (28.92° ± 7.43°, range 20°–51°, *p* = 0.007).	S. Zhou (2012)
***IGF-1* (Insuline growth factor–1)**	rs5742612-TT genotype	IGF-I has a pivotal role in bone growth determining different skeletal growth	Sensitivity: 88% Specificity: 22% Positive predictive value: 57% Negative predictive value: 61%	0.04	Cobb’s angle is higher in patients with TT genotype (Mean Cobb’s angle: 38.1° in TT vs. 35.9° in TC vs. 33.2° in CC group).	Y. Yeung (2006)
***IGF-1* (Insuline growth factor–1)**	rs5742612-GG genotype	IGF-I has a pivotal role in bone growth determining different skeletal growth	NS	0.01	IGF1polymorphism rs5742612 significantly differs among controls, high-risk, and low-risk groups.	S. Moon (2013)
***MATN-1* (Matrillin 1)**	rs1149048-allele G	Matrilin-1 is secreted primarily by chondrocytes and has a role in the assembly of cartilage. It has been confirmed that matrilin-1 has an important function in the organization of chondrocyte into distinct zones of growth plate. Disturbance of the chondrocyte zonal distribution could lead to musculoskeletal disorders, such as scoliosis.	OR = 1.35 within 95% confidence interval (CI): 1.14–1.61	0.02	The mean maximal Cobb angle of patients with Rs1149048 SNPs is genotype GG: 37.91 ± 17.081, Genotype AA: 33.88 ± 14.681, Genotype AG: 32.25 ± 12. 421. Genotype GG develop a larger Cobb angle than those with genotype AA with a statistically significant difference. The tagSNP rs1149048 polymorphism in the MATN1 promoter region is associated with both susceptibility and disease progression in AIS.	Z. Chen (2009)
***TPH-1* (Trypophan hydroxylase 1)**	rs10488682-Genotype AT and A allele	Tryptophan hydroxylases catalyze the biopterin-dependent monooxygenation of tryptophan to 5- hydroxytryptophan to (5-HTP), which is subsequently decarboxylated to form the neurotransmitter serotonin (5-hydroxytryptamine or 5-HT). It is the rate-limiting enzyme in the biosynthesis of serotonin.	Sensitivity: 51% Specificity: 82% OR = 2.289 within 95% Confidence Interval (CI): 1.18–4.43	0.002 0.033	Statistically significant differences between the two groups (progression vs. non-progression) in SNP rs10488682 in THP-1: genotype AT (33.3 vs. 13.0%), allele A (16.7 vs. 9.6%). Allele A of THP-1 could be considered a risk factor leading to progression of AIS curve.	L. Xu (2011)
***NFT3* (Neurothropin 3)**	rs11063714-AA genotype	Scoliosis has developed in mice with NTF3 deficiency in previous studies. Increased expression of NTF3 mRNA was detected in the paravertebral muscle in AIS.	Sensitivity: 43% Specificity: 82% Positive predictive value: 56% Negative predictive value: 72%	<0.05	For rs11063714 SNP, AIS patients with AA genotype had a significantly lower mean maximum Cobb angle than the patients with AG or GG genotypes, respectively: 25.45 ± 8.69 vs. 32.32 ± 13.36 vs. 34.26 ± 17.41. For rs11063714 SNP, there was a significantly higher successful ratio of brace treatment in AA genotype compared to GG genotype, respectively: 81.6% vs. 57.7%.	Y. Qiu (2012)
***LBX1* (Ladybird homebox 1)**	rs11190870-TT genotype	LBX1 has an important role in developmental processes. This gene is expressed in the central nervous system and skeletal muscle	OR = 1.51 within 95% Confidence interval (CI): 1.33–1.71	<0.001	AIS patients with TT genotype of rs11190870 had a larger Cobb angle than those with TC or CC genotype (50.8% vs. 25%; *p* < 0.001)	H. Jiang (2013)
***TGFB1* (transforming growth factor beta 1)**	Rs1800469 Rs1800471	TGFβ-1 protein triggers chemical signals that regulate various cell activities inside the cell, including the growth and division (proliferation) of cells, the maturation of cells to carry out specific functions (differentiation), cell movement (motility), and controlled cell death (apoptosis)	OR = 3.78 within 95% Confidence interval (CI): 1.42–10.05	0.038	Kruskal–Wallis analysis of variance revealed the relationship between the SNP C-509T of the TGFB1 gene and the curve severity in females with AIS (Kruskal–Wallis statistic = 6.50)	I. Ryzhkov (2013)
***LAPTM4B* (Lysosomal-associated transmembrane protein 4 beta)**	rs2449539	LAPTM4B is required for optimal lysosomal function. It blocks EGF-stimulated EGFR intraluminal sorting and degradation. Conversely, by binding with the phosphatidylinositol 4,5-bisphosphate, it regulates its PIP5K1C interaction, inhibits HGS ubiquitination, and relieves LAPTM4B inhibition of EGFR degradation	NS	0.014	LAPTM4B (lysosomal-associated transmembrane protein 4β) polymorphism rs2449539 significantly differs among the lower and high-risk groups. TT genotype most frequent in high-risk group and TC genotype in control group.	S. Moon (2013)
***FNB1/2* (Fibrillin 1 and 2)**	Rage damaging variants	Fibrillin mutations are the main mutated protein causing Marfan syndrome. This mutation usually interferes with the assembly of microfibrils resulting in a dominant, negative mechanism.	OR = 3.5 within 95% Confidence interval (CI): 1.6–7.3	0.026	The average spinal curve in AIS cases with a rare FBN1 or FBN2 variant was 50.58°, compared with 42.18° in cases with no fibrillin variant. This indicates that FBN1 and FBN2 variants could serve as prognostic genetic markers to predict scoliosis progression.	J Buchan (2014)
***FNB1* (Fibrillin 1)**	106 SNPs studied	Fibrillin mutations are the main mutated protein causing Marfan syndrome. This mutation usually interferes with the assembly of microfibrils resulting in a dominant, negative mechanism.	OR = 1.78 within 95% Confidence interval (CI): 0.59–2.53	0.02	The decreased expression level of FBN1 was remarkably correlated with the curve severity. The functional role of FBN1 in the progression of the AIS is worthy of further investigation.	F. Sheng (2018)
**BCN 2 (Basonuclein 2)**	rs10738445-Genotype CC	This gene encodes a conserved zinc finger protein. The encoded protein functions in skin color saturation. Mutations in this gene are associated with facial pigmented spots. This gene is also associated with susceptibility to adolescent idiopathic scoliosis	OR = 1.24 within 95% Confidence interval (CI): 1.01–1.54	0.01	AIS patients were found to have significantly higher expression of the BNC2 as compared to controls. Moreover, AIS patients with genotype CC have larger Cobb angle than those with genotype TT (41.3 ± 13.5 vs. 35.4 ± 14.1).	L. Xu (2017)
***TIMP2* (Tissue inhibitor of metalloproteinase 2)**	rs2277700, rs11077401, rs2376999, and rs4789934	The proteins encoded by this gene family are natural inhibitors of the matrix metalloproteinases (MMP), a group of peptidases involved in degradation of the extracellular matrix.	rs2277700-allele G: OR = 0.34 within 95%, Confidence interval (CI):0.16–0.74 rs11077401-allele T: OR = 0.13 within 95%, Confidence interval (CI):0.05–0.31 rs2376999-allele T OR = 0.37, Confidence interval (CI): 0.15–0.99 Rs478934-allele T OR = 0.21, Confidence Interval (CI): 0.04–1	rs2277700-allele G: <0.01 rs11077401-allele T: <0.01 rs2376999-allele T: =0.04 Rs478934-allele T: =0.048	Four of the polymorphisms (rs2277700, rs11077401, rs2376999, and rs4789934) showed non-equal distributions either in genotype or/and allele distributions in the patients of different progression rates.	M. Andrusiewicz (2019)
***SOX9* (SRY-box transcription factor 9 SOX9)**	rs12946942-recessive allele	It is expressed by proliferating, but not hypertrophic chondrocytes, which is essential for the differentiation of precursor cells into chondrocytes	OR = 1.36 within 95% Confidence Interval (CI): 1.25–1.49	<0.01	The recessive allele of rs12946942 SNP showed significant association in severe AIS patients.	K. Takeda (2019)
***CHD7* (chromodomain helicase DNA binding protein 7)**	rs1017861-GG and AA alleles	CHD7 is essential for the formation of multipotent migratory neural crest and their ability to migrate throughout the body.	Rs1017861 GG: OR = 3.3 within 95% Confidence Interval (CI): 0.9–12.7 Rs1017861 AA: OR = 0.4 within 95% Confidence Interval (CI): 0.2–0.6	Rs1017861 GG: 0.0001 Rs1017861 AA: 0.002	Two polymorphisms, rs1017861 and rs4738813, were associated with curve severity and progression rate.	K. Borysiak (2020)
***MIR4300* (microRNA4300 gene)**	Rs1828853	MIR4300HG is highly expressed in spinal cord, brain, skeletal muscle, salivary gland, and epithelial cells in various tissues and sperm	OR = 1.56 within 95% Confidence Interval (CI):1.35–1.80	<0.001	MIR4300 host gene SNP rs1828853 showed association with progression of AIS.	Y. Ogura (2017)
***MIR4300* HG (microRna 4300 gene)**	rs35333564-allele G	RNAs are involved in post-transcriptional regulation of gene expression in multicellular organisms by affecting both the stability and translation of mRNAs	rs35333564-allele G: OR = 1.339 within 95% Confidence interval (CI): 1.07–1.67	0.01	Significant difference between two groups regarding both genotype frequency (3.1% vs. 1.3%, *p* = 0.025) and minor allele frequency (17.5% vs. 13.7%, *p* = 0.011) of rs35333564 in MIR4300 gene.	Y. Wang (2021)

**Table 3:** Epigenetic factors associated with AIS progression (NS = non-specified, OR = odds ratio, AUC = area under the curve).

Epigenetic Marker	Technique	Biological Sample	Molecular Pathway	Sensitivity/Specificity	*p*-Value	Results	Reference
**cg01374129 demethylation status correlates with disease progression (*HAS2* as candidate gene).**	Whole-exome sequencing and quantitative DNA methylation analysis by Massarray	Peripheral blood cell DNA of AIS discordant monozygotic twin pairs	The Wnt/β-catenin signaling pathway plays a prominent role in maintaining cellular homeostasis, bone formation, and remodeling.	Sensitivity: 76.4%, Specificity: 85.6% AUC = 0.827 within 95% Confidence interval (CI): 0.780–0.876	<0.0001	Methylation level of cg01374129 site (Has2 gene) was significantly lower in the progression group than in the non-progression group. Cg01374129 methylation as biomarker achieved a sensitivity of 76.4% and a specificity of 85.6% in differentiating patients with and without curve progression.	Y. Meng (2018)
**cg02477677, cg12922161, cg08826461, and cg16382077** **methylation associated with curve severity (*WNT10A* and *NPY* as candidate genes)**	Array-based genome-wide methylation analysis	Peripheral blood cell DNA of AIS monozygotic twin pairs	WNT signaling pathway relevant for bone formation and remodeling; neuropeptide Y (NPY), regulator of bone and energy homeostasis	NS	=0.494, FDR adjusted *p*-value = 0.41329	Hypomethylation of four CpG sites was associated with curve severity (cg02477677, cg12922161, cg08826461, and cg1638077). Annotation of two of the regions implicated the NPY gene on chr. 7 and the WNT10A gene on chr. 2	P. Carry (2021)
**Overexpression of *miR145* of Wnt/β-catenin signaling pathway**	Array-based miRNA expression analysis	Iliac crest bone tissue cells of AIS patients and serum	WNT signaling pathway relevant to bone formation and remodeling	Sensitivity 72.7% Specificity 90% AUC = 0.93; within 95% Confidence Interval (CI): 0.88–0.98	<0.05	Significant negative correlations between circulating miR-145 and serum sclerostin, osteopontin, and osteoprotegerin in AIS patients and not in control group. Aberrant miRNA expression may contribute to low bone mass and affect osteocyte function, with possible involvement in AIS pathogenesis.	J. Zhang (2018)
***COMP* promoter methylation associated with curve severity**	Pyrosequencing	Peripheral blood cell DNA of AIS patients and controls	COMP (cartilage oligomerix matrix protein) belongs to the trombospondin gene family and is a marker of cartilage turnover.	NS	<0.001	The methylation level of five CpGs in the COMP promoter was significantly correlated with Cobb angle of the main curve and chronological age (*p* < 0.0001).	S. Mao (2018)
***PITX1* promoter methylation** **associated with Cobb angle**	Pyrosequencing	Peripheral blood cell DNA of AIS patients and controls	PITX1 is a member of the RIEG/PITX homeobox transcription factor family, involved in organ development. Mutations in this gene have been associated with various bone-related diseases.	NS	<0.001	The methylation level of 6 CpG sites in PITX1 promoter was significantly associated with Cobb angle of the main curve. The comparative analysis showed significant difference in age (*p* = 0.021) and Cobb angle of the main curve (*p* = 0.0001) between AIS groups with positive and negative methylation.	B. Shi (2018)
***PCDH10* promoter methylation level associated with Cobb angle**	Pyrosequencing	Peripheral blood cell DNA of controls and AIS patients	protocadherin10 (PCDH10) gene, involved in immune process and Wnt	NS	<0.001	AIS patients were associated with high Higher DNA methylation level and low gene expression of PCDH10 gene rather than normal controls. The high methylation level indicated high Cobb angle of major curves in AIS. The abnormal DNA methylation may widely exist and serve as a potential mechanism for AIS progression. The average methylation level was 4.32 ± 0.73 in AIS patients and 3.14 ± 0.97 in healthy controls (*p* < 0.001). Besides, the PCDH10 gene expression was 0.23 ± 0.04 in AIS patients and 0.36 ± 0.08 in normal controls (*p* < 0.001).	B. Shi (2019)
***H19* downregulation and *ADIPOQ* upregulation in concave-sided muscle correlate positively with curve severity and age at initiation.**	RNA-seq	Paravertebral muscle concave and convex muscles of AIS patients	ADIPOQ (PARR signaling pathway, gene encoding for adiponectin) and H19 (long non-coding RNA generating miR-675-5p and miR-675-3p) H19 can promote skeletal muscle differentiation and regeneration and regulate glucose metabolism.	NS	<0.001 0.011 <0.001 0.039	RNA-seq revealed transcriptomic differences between two sides of paravertebral muscle in AIS patients. This implies that transcriptomic differences caused by epigenetic factors in affected individuals may account for the structural and functional imbalance of paravertebral muscle, which can expand the understanding of this disease progression. Comparing features of clinical characteristics, such as the magnitude of spinal curve, age at menarche, body mass index and age at initiation, between different samples with different ADIPOQ and H19 expression patterns. The relative expression difference of H19 (concave–convex) was significantly correlated with Cobb’s angle (r = 0.638, *p* < 0.001) and age at initiation (r = − 0.295, *p* = 0.011), and the relative expression difference of ADPOQ mRNA (concave-convex) was also significantly correlated with spinal curve (r = − 0.4926, *p* < 0.001) and age at initiation (r = 0.230, *p* = 0.039). These data suggest an important role of H19 and ADIPOQ in the onset or progression of scoliosis.	H. Jiang (2018)
**T-DMR1 and T-DMR2 regions of *ESR1* gene methylation associated with AIS severity**	Pyrosequencing	Paraspinal superficial and deep muscles of AIS patients	Estrogen receptor	NS	0.02 0.04 0.04 0.05	In the deep paravertebral muscle, the methylation level within the ESR1 T-DMR2 region on the concave side of the curvature was significantly different between groups of patients with a Cobb angle >70° or <70° at four CpG sites: CPG2, CPG3, CPG4, and CPG6. No differences were observed in T-DMR1 methylation levels between groups of patients with Cobb angles <70° and >70°.	P. Janusz, (2021)
***miR-151a-3p* (targeting *GREM1*)**	NGS Small RNA sequencing	Cell-free RNA from peripheral blood plasma of severe and mild AIS patients and controls	Skeletal homeostasis	AUC = 0.885 within 95% Confidence Interval (CI): 0.815–0.936	<0.05	miR-151a-3p and GREM1 expression significantly correlated with severe AIS curves. Plasma miR-151a-3p might serve as a biomarker for severe AIS. The overexpression of miR-151a-3p may contribute to the progression of scoliosis via inhibition of GREM1 expression in osteoblasts to interrupt bone homeostasis. Finally, relatively lower methylation levels of the promoter of miR-151a-3p might explain high miR-151a-3p levels. This may provide a new biomarker for the early detection of AIS and increase our understanding of the progression of AIS.	Wang (2020)

## Data Availability

Not applicable.

## Supplementary Materials

The following supporting information can be downloaded at: https://www.mdpi.com/article/10.3390/ijms23115914/s1.

## References

[1] Faldini C., Perna F., Geraci G., Pardo F., Mazzotti A., Pilla F., Ruffilli A. (2018). Triplanar Correction of Adolescent Idiopathic Scoliosis by Asymmetrically Shaped and Simultaneously Applied Rods Associated with Direct Vertebral Rotation: Clinical and Radiological Analysis of 36 Patients. Eur. Spine J..

[2] Barile F., Ruffilli A., Manzetti M., Fiore M., Panciera A., Viroli G., Faldini C. (2021). Resumption of Sport after Spinal Fusion for Adolescent Idiopathic Scoliosis: A Review of the Current Literature. Spine Deform..

[3] Konieczny M.R., Senyurt H., Krauspe R. (2013). Epidemiology of Adolescent Idiopathic Scoliosis. J. Child. Orthop..

[4] Lonstein J.E. (1994). Adolescent Idiopathic Scoliosis. Lancet.

[5] Lonstein J.E., Carlson J.M. (1984). The Prediction of Curve Progression in Untreated Idiopathic Scoliosis during Growth. J. Bone Jt. Surg. Ser. A.

[6] Pérez-Machado G., Berenguer-Pascual E., Bovea-Marco M., Rubio-Belmar P.A., García-López E., Garzón M.J., Mena-Mollá S., Pallardó F.V., Bas T., Viña J.R. (2020). From Genetics to Epigenetics to Unravel the Etiology of Adolescent Idiopathic Scoliosis. Bone.

[7] García-Cano E., Arámbula Cosío F., Duong L., Bellefleur C., Roy-Beaudry M., Joncas J., Parent S., Labelle H. (2018). Prediction of Spinal Curve Progression in Adolescent Idiopathic Scoliosis Using Random Forest Regression. Comput. Biol. Med..

[8] Zhu Z., Tang N.L.S., Xu L., Qin X., Mao S., Song Y., Liu L., Li F., Liu P., Yi L. (2015). Genome-Wide Association Study Identifies New Susceptibility Loci for Adolescent Idiopathic Scoliosis in Chinese Girls. Nat. Commun..

[9] Londono D., Kou I., Johnson T.A., Sharma S., Ogura Y., Tsunoda T., Takahashi A., Matsumoto M., Herring J.A., Lam T.P. (2014). A Meta-Analysis Identifies Adolescent Idiopathic Scoliosis Association with LBX1 Locus in Multiple Ethnic Groups. J. Med. Genet..

[10] Xu L., Huang S., Qin X., Mao S., Qiao J., Qian B.P., Qiu Y., Zhu Z. (2015). Investigation of the 53 Markers in a DNA-Based Prognostic Test Revealing New Predisposition Genes for Adolescent Idiopathic Scoliosis. Spine.

[11] Xu L., Wu Z., Xia C., Tang N., Cheng J.C.Y., Qiu Y., Zhu Z. (2019). A Genetic Predictive Model Estimating the Risk of Developing Adolescent Idiopathic Scoliosis. Curr. Genom..

[12] Liberati A., Altman D.G., Tetzlaff J., Mulrow C., Gøtzsche P.C., Ioannidis J.P.A., Clarke M., Devereaux P.J., Kleijnen J., Moher D. (2009). The PRISMA Statement for Reporting Systematic Reviews and Meta-Analyses of Studies That Evaluate Healthcare Interventions: Explanation and Elaboration. BMJ.

[13] OCEBM Levels of Evidence Working Group “The Oxford 2011 Levels of Evidence”. Oxford Centre for Evidence-Based Medicine. https://www.cebm.net/wp-content/uploads/2014/06/CEBM-Levels-of-Evidence-2.1.pdf.

[14] Sideri S., Papageorgiou S.N., Eliades T. (2018). Registration in the International Prospective Register of Systematic Reviews (PROSPERO) of Systematic Review Protocols Was Associated with Increased Review Quality. J. Clin. Epidemiol..

[15] NIH National Heart, Lung and Blood Institute Study Quality Assessment Tools. Https://Www.Nhlbi.Nih.Gov/Health-Topics/Assessing-Cardiovascular-Risk.

[16] Inoue M., Minami S., Nakata Y., Takaso M., Otsuka Y., Kitahara H., Isobe K., Kotani T., Maruta T., Moriya H. (2002). Prediction of Curve Progression in Idiopathic Scoliosis from Gene Polymorphic Analysis. Stud. Health Technol. Inform..

[17] Inoue M., Minami S., Nakata Y., Kitahara H., Otsuka Y., Isobe K., Takaso M., Tokunaga M., Nishikawa S., Maruta T. (2002). Association between Estrogen Receptor Gene Polymorphisms and Curve Severity of Idiopathic Scoliosis. Spine.

[18] Yilmaz H., Zateri C., Uludag A., Bakar C., Kosar S., Ozdemir O. (2012). Single-Nucleotide Polymorphism in Turkish Patients with Adolescent Idiopathic Scoliosis: Curve Progression Is Not Related with MATN-1, LCT C/T-13910, and VDR BsmI. J. Orthop. Res..

[19] Ryzhkov I.I., Borzilov E.E., Churnosov M.I., Ataman A.V., Dedkov A.A., Polonikov A.V. (2013). Transforming Growth Factor Beta 1 Is a Novel Susceptibility Gene for Adolescent Idiopathic Scoliosis. Spine.

[20] Jiang H., Qiu X., Dai J., Yan H., Zhu Z., Qian B., Qiu Y. (2013). Association of Rs11190870 near LBX1 with Adolescent Idiopathic Scoliosis Susceptibility in a Han Chinese Population. Eur. Spine J..

[21] Moon E.S., Kim H.S., Sharma V., Park J.O., Lee H.M., Moon S.H., Chong H.S. (2013). Analysis of Single Nucleotide Polymorphism in Adolescent Idiopathic Scoliosis in Korea: For Personalized Treatment. Yonsei Med. J..

[22] Ogura Y., Takahashi Y., Kou I., Nakajima M., Kono K., Kawakami N., Uno K., Ito M., Minami S., Yanagida H. (2013). A Replication Study for Association of 5 Single Nucleotide Polymorphisms with Curve Progression of Adolescent Idiopathic Scoliosis in Japanese Patients. Spine.

[23] Buchan J.G., Alvarado D.M., Haller G.E., Cruchaga C., Harms M.B., Zhang T., Willing M.C., Grange D.K., Braverman A.C., Miller N.H. (2014). Rare Variants in FBN1 and FBN2 Are Associated with Severe Adolescent Idiopathic Scoliosis. Hum. Mol. Genet..

[24] Kotwicki T., Janusz P., Andrusiewicz M., Chmielewska M., Kotwicka M. (2014). Estrogen Receptor 2 Gene Polymorphism in Idiopathic Scoliosis. Spine.

[25] Roye B.D., Wright M.L., Matsumoto H., Yorgova P., McCalla D., Hyman J.E., Roye D.P., Shah S.A., Vitale M.G. (2014). An Independent Evaluation of the Validity of a DNA-Based Prognostic Test for Adolescent Idiopathic Scoliosis. J. Bone Jt. Surg. Am. Vol..

[26] Tang Q.L., Julien C., Eveleigh R., Bourque G., Franco A., Labelle H., Grimard G., Parent S., Ouellet J., Mac-Thiong J.M. (2015). A Replication Study for Association of 53 Single Nucleotide Polymorphisms in ScoliScore Test with Adolescent Idiopathic Scoliosis in French-Canadian Population. Spine.

[27] Bohl D.D., Telles C.J., Ruiz F.K., Badrinath R., DeLuca P.A., Grauer J.N. (2016). A Genetic Test Predicts Providence Brace Success for Adolescent Idiopathic Scoliosis When Failure Is Defined as Progression to > 45 Degrees. Clin. Spine Surg..

[28] Yeung H.Y., Tang N.L., Lee K.M., Ng B.K.W., Hung V.W.Y., Kwok R., Guo X., Qin L., Cheng J.C.Y. (2006). Genetic Association Study of Insulin-like Growth Factor-I (IGF-I) Gene with Curve Severity and Osteopenia in Adolescent Idiopathic Scoliosis. Stud. Health Technol. Inform..

[29] Xu L., Qin X., Sun W., Qiao J., Qiu Y., Zhu Z. (2016). Replication of Association between 53 Single-Nucleotide Polymorphisms in a DNA-Based Diagnostic Test and AIS Progression in Chinese Han Population. Spine.

[30] Ogura Y., Kou I., Takahashi Y., Takeda K., Minami S., Kawakami N., Uno K., Ito M., Yonezawa I., Kaito T. (2017). A Functional Variant in MIR4300HG, the Host Gene of MicroRNA MIR4300 Is Associated with Progression of Adolescent Idiopathic Scoliosis. Hum. Mol. Genet..

[31] Xu L., Xia C., Qin X., Sun W., Tang N.L.S., Qiu Y., Cheng J.C.Y., Zhu Z. (2017). Genetic Variant of BNC2 Gene Is Functionally Associated with Adolescent Idiopathic Scoliosis in Chinese Population. Mol. Genet. Genomics.

[32] Takahashi Y., Kou I., Ogura Y., Miyake A., Takeda K., Nakajima M., Minami S., Kawakami N., Uno K., Ito M. (2018). A Replication Study for the Association of Rs11190870 With Curve Severity in Adolescent Idiopathic Scoliosis in Japanese. Spine.

[33] Man G.C.W., Tang N.L.S., Chan T.F., Lam T.P., Li J.W., Ng B.K.W., Zhu Z., Qiu Y., Cheng J.C.Y. (2019). Replication Study for the Association of GWAS-Associated Loci with Adolescent Idiopathic Scoliosis Susceptibility and Curve Progression in a Chinese Population. Spine.

[34] Meng Y., Lin T., Liang S., Gao R., Jiang H., Shao W., Yang F., Zhou X. (2018). Value of DNA Methylation in Predicting Curve Progression in Patients with Adolescent Idiopathic Scoliosis. EBioMedicine.

[35] Zhang J., Chen H., Leung R.K.K., Choy K.W., Lam T.P., Ng B.K.W., Qiu Y., Feng J.Q., Cheng J.C.Y., Lee W.Y.W. (2018). Aberrant MiR-145-5p/b-Catenin Signal Impairs Osteocyte Function in Adolescent Idiopathic Scoliosis. FASEB J..

[36] Mao S., Qian B., Shi B., Zhu Z., Qiu Y. (2018). Quantitative Evaluation of the Relationship between COMP Promoter Methylation and the Susceptibility and Curve Progression of Adolescent Idiopathic Scoliosis. Eur. Spine J..

[37] Sheng F., Xia C., Xu L., Qin X., Tang N.L.S., Qiu Y., Cheng J.C.Y., Zhu Z. (2019). New Evidence Supporting the Role of FBN1 in the Development of Adolescent Idiopathic Scoliosis. Spine.

[38] Shi B., Xu L.L., Mao S., Xu L.L., Liu Z., Sun X., Zhu Z., Qiu Y. (2018). Abnormal PITX1 Gene Methylation in Adolescent Idiopathic Scoliosis: A Pilot Study. BMC Musculoskelet. Disord..

[39] Tang N.L.S., Yeung H.Y., Lee K.M., Hung V.W.Y., Cheung C.S.K., Ng B.K.W., Kwok R., Guo X., Qin L., Cheng J.C.Y. (2006). A Relook into the Association of the Estrogen Receptor α Gene (PvuII, XbaI) and Adolescent Idiopathic Scoliosis: A Study of 540 Chinese Cases. Spine.

[40] Jiang H., Yang F., Lin T., Shao W., Meng Y., Ma J., Wang C., Gao R., Zhou X. (2018). Asymmetric Expression of H19 and ADIPOQ in Concave/Convex Paravertebral Muscles Is Associated with Severe Adolescent Idiopathic Scoliosis. Molecular.

[41] Andrusiewicz M., Harasymczuk P., Janusz P., Biecek P., Żbikowska A., Kotwicka M., Kotwicki T. (2019). TIMP2 Polymorphisms Association with Curve Initiation and Progression of Thoracic Idiopathic Scoliosis in the Caucasian Females. J. Orthop. Res..

[42] Jiang H., Liang S., He K., Hu J., Xu E., Lin T., Meng Y., Zhao J., Ma J., Gao R. (2020). Exome Sequencing Analysis Identifies Frequent Oligogenic Involvement and FLNB Variants in Adolescent Idiopathic Scoliosis. J. Med. Genet..

[43] Takeda K., Kou I., Otomo N., Grauers A., Fan Y.H., Ogura Y., Takahashi Y., Momozawa Y., Einarsdottir E., Kere J. (2019). A Multiethnic Meta-Analysis Defined the Association of Rs12946942 with Severe Adolescent Idiopathic Scoliosis. J. Hum. Genet..

[44] Shi B., Mao S., Xu L., Li Y., Sun X., Liu Z., Zhu Z., Qiu Y. (2020). Quantitation Analysis of PCDH10 Methylation in Adolescent Idiopathic Scoliosis Using Pyrosequencing Study. Spine.

[45] Wang Y., Zhang H., Yang G., Xiao L., Li J., Guo C. (2020). Dysregulated Bone Metabolism Is Related to High Expression of MiR-151a-3p in Severe Adolescent Idiopathic Scoliosis. BioMed Res. Int..

[46] Borysiak K., Janusz P., Andrusiewicz M., Chmielewska M., Kozinoga M., Kotwicki T., Kotwicka M. (2020). CHD7 Gene Polymorphisms in Female Patients with Idiopathic Scoliosis. BMC Musculoskelet. Disord..

[47] Wang Y., Dai Z., Wu Z., Feng Z., Liu Z., Sun X., Xu L., Qiu Y., Zhu Z. (2021). Genetic Variant of MIR4300HG Is Associated with Progression of Adolescent Idiopathic Scoliosis in a Chinese Population. J. Orthop. Surg. Res..

[48] Carry P.M., Terhune E.A., Trahan G.D., Vanderlinden L.A., Wethey C.I., Ebrahimi P., McGuigan F., Åkesson K., Hadley-miller N. (2021). Severity of Idiopathic Scoliosis Is Associated with Differential Methylation: An Epigenome-Wide Association Study of Monozygotic Twins with Idiopathic Scoliosis. Genes.

[49] Janusz P., Chmielewska M., Andrusiewicz M., Kotwicka M., Kotwicki T. (2021). Methylation of Estrogen Receptor 1 Gene in the Paraspinal Muscles of Girls with Idiopathic Scoliosis and Its Association with Disease Severity. Genes.

[50] Chen Z., Tang N.L.S., Cao X., Qiao D., Yi L., Cheng J.C.Y., Qiu Y. (2009). Promoter Polymorphism of Matrilin-1 Gene Predisposes to Adolescent Idiopathic Scoliosis in a Chinese Population. Eur. J. Hum. Genet..

[51] Zhao D., Xing Qiu G., Peng Wang Y., Guo Zhang J., Xiong Shen J., Hong Wu Z., Wang H. (2009). Association of Calmodulin1 Gene Polymorphisms with Susceptibility to Adolescent Idiopathic Scoliosis. Orthop. Surg..

[52] Ward K., Ogilvie J.W., Singleton M.V., Chettier R., Engler G., Nelson L.M. (2010). Validation of DNA-Based Prognostic Testing to Predict Spinal Curve Progression in Adolescent Idiopathic Scoliosis. Spine.

[53] Xu L., Qiu X., Sun X., Mao S., Liu Z., Qiao J., Qiu Y. (2011). Potential Genetic Markers Predicting the Outcome of Brace Treatment in Patients with Adolescent Idiopathic Scoliosis. Eur. Spine J..

[54] Qiu Y., Mao S.H., Qian B.P., Jiang J., Qiu X.S., Zhao Q., Liu Z. (2012). A Promoter Polymorphism of Neurotrophin 3 Gene Is Associated with Curve Severity and Bracing Effectiveness in Adolescent Idiopathic Scoliosis. Spine.

[55] Zhou S., Qiu X.S., Zhu Z.Z., Wu W.F., Liu Z., Qiu Y. (2012). A Single-Nucleotide Polymorphism Rs708567 in the IL-17RC Gene Is Associated with a Susceptibility to and the Curve Severity of Adolescent Idiopathic Scoliosis in a Chinese Han Population: A Case-Control Study. BMC Musculoskelet. Disord..

[56] Chen S., Anderson M.V., Cheng W.K., Wongworawat M.D. (2009). Diabetes Associated with Increased Surgical Site Infections in Spinal Arthrodesis. Proceedings of the 2008 Meeting of the Musculoskeletal Infection Society.

[57] Giustina A., Mazziotti G., Canalis E. (2008). Growth Hormone, Insulin-Like Growth Factors, and the Skeleton. Endocr. Rev..

[58] Takahashi Y., Matsumoto M., Karasugi T., Watanabe K., Chiba K., Kawakami N., Tsuji T., Uno K., Suzuki T., Ito M. (2011). Lack of Association between Adolescent Idiopathic Scoliosis and Previously Reported Single Nucleotide Polymorphisms in MATN1, MTNR1B, TPH1, and IGF1 in a Japanese Population. J. Orthop. Res..

[59] Vidal O., Kindblom L.G., Ohlsson C. (1999). Expression and Localization of Estrogen Receptor-β in Murine and Human Bone. J. Bone Miner. Res..

[60] Ramirez F., Pereira L., Zhang H., Lee B. (1993). The Fibrillin-Marfan Syndrome Connection. Bioessays.

[61] Reik W. (2007). Stability and Flexibility of Epigenetic Gene Regulation in Mammalian Development. Nat..

[62] Oh E.S., Petronis A. (2021). Origins of Human Disease: The Chrono-Epigenetic Perspective. Nat. Rev. Genet..

[63] Chmielewska M., Janusz P., Andrusiewicz M., Kotwicki T., Kotwicka M. (2020). Methylation of Estrogen Receptor 2 (ESR2) in Deep Paravertebral Muscles and Its Association with Idiopathic Scoliosis. Sci. Rep..

[64] Ylikoski M. (2005). Growth and Progression of Adolescent Idiopathic Scoliosis in Girls. J. Pediatr. Orthop. B.

[65] Saccomani L., Vercellino F., Rizzo P., Becchetti S. (1998). Adolescents with Scoliosis: Psychological and Psychopathological Aspects. Minerva Pediatr..

[66] Asher M.A., Min Lai S., Burton D.C. (2000). Further Development and Validation of the Scoliosis Research Society (SRS) Outcomes Instrument. Spine.

[67] Luan F.J., Wan Y., Mak K.C., Ma C.J., Wang H.Q. (2020). Cancer and Mortality Risks of Patients with Scoliosis from Radiation Exposure: A Systematic Review and Meta-Analysis. Eur. Spine J..

[68] Simony A., Hansen E.J., Christensen S.B., Carreon L.Y., Andersen M.O. (2016). Incidence of Cancer in Adolescent Idiopathic Scoliosis Patients Treated 25 Years Previously. Eur. Spine J..

[69] Murphy R.F., Mooney J.F. (2016). Complications Following Spine Fusion for Adolescent Idiopathic Scoliosis. Curr. Rev. Musculoskelet. Med..

[70] Carreon L.Y., Puno R.M., Lenke L.G., Richards B.S., Sucato D.J., Emans J.B., Erickson M.A. (2007). Non-Neurologic Complications Following Surgery for Adolescent Idiopathic Scoliosis. J. Bone Jt. Surg. Ser. A.

[71] Menger R.P., Kalakoti P., Pugely A.J., Nanda A., Sin A. (2017). Adolescent Idiopathic Scoliosis: Risk Factors for Complications and the Effect of Hospital Volume on Outcomes. Neurosurg. Focus.

