# Supplementary material for: Epigenetic and Genetic Factors Related to Curve Progression in Adolescent Idiopathic Scoliosis: A Systematic Scoping Review of the Current Literature
Source: Int J Mol Sci. 2022 May 25;23(11):5914. doi: 10.3390/ijms23115914 (PMC9180299; doi:10.3390/ijms23115914)
Supplement: Supplementary file 1 [file ijms-23-05914-s001.zip › Table S1 - Search strategy table.pdf]

Table S1: Search strategy table

	Concept 1	Concept 2	Concept 3	Concept 4
**Key concepts**	Adolescent Idiopathic Scoliosis	Curve progression	Curve severity	Genetic and\or epigenetic variants
**Free text terms / natural language terms**<br>(Synonyms, UK/US terminology, medical/laymen's terms, acronyms/abbreviations, drug brands, more narrow search terms)	AIS<br>Idiopathic scoliosis<br>Scoliosis of the adolescent	Curve worsening<br>Curve evolution	Curve magnitude<br>High Cobb angle	Single nucleotide Polymorphism (SNP)<br>Mutation<br>Genetic and\or epigenetic variants
**Subject fields**<br>(Title, abstract, keyword)	Title, abstract, keyword	Title, abstract, keyword	Title, abstract, keyword	Title, abstract, keyword
