# Supplementary material for: Epigenetic and Genetic Factors Related to Curve Progression in Adolescent Idiopathic Scoliosis: A Systematic Scoping Review of the Current Literature
Source: Int J Mol Sci. 2022 May 25;23(11):5914. doi: 10.3390/ijms23115914 (PMC9180299; doi:10.3390/ijms23115914)
Supplement: Supplementary file 1 [file ijms-23-05914-s001.zip › Table S2 - Quality assessment.pdf]

Table S2: Studies were assessed using the NIH quality assessment tool Quality was rated as 0 for poor (0–3 out of 9 questions), 1 for fair (4–6 out of 9 questions), or 2 for good (7–9 out of 9 questions).

Studies	Was the study question or objective clearly stated?	Was the study population clearly and fully described, including a case definition?	Were the cases consecutive?	Were the subjects comparable?	Was the intervention clearly described?	Were the outcome measures clearly defined, valid, reliable, and implemented consistently across all study participants?	Was the length of follow-up adequate?	Were the statistical methods well-described?	Were the results well-described?	Quality summary
**M. Inoue (2002)**	✔	✔	✔	✔	✔	✔	✔	✗	✔	**2**
**M. Inoue (2002)**	✔	✔	✔	✔	✔	✔	✔	✗	✔	**2**

**Y. Yeung (2006)**	✔	✗	✗	✔	✗	✔	✔	✔	✔	**1**
**N. Tang (2006)**	✔	✔	✗	✔	✔	✔	✔	✔	✔	**2**
**Z.Chen (2009)**	✔	✗	✔	✔	✔	✔	✔	✔	✔	**2**

**D. Zhao (2009)**	✔	✔	✔	✔	✔	✔	✗	✗	✔	**2**
**K. Ward (2010)**	✔	✗	✔	✔	✔	✔	✗	✔	✔	**2**
**L. Xu (2011)**	✔	✔	✔	✔	✔	✔	✗	✔	✔	**2**

**Y. Qiu (2012)**	✔	✗	✔	✔	✔	✔	✔	✔	✔	**2**
**S. Zhou (2012)**	✔	✗	✔	✔	✔	✔	✗	✔	✔	**2**
**H.Yilmaz (2012)**	✔	✗	✔	✔	✔	✔	✗	✗	✔	**1**

**I. Ryzhkov (2013)**	✔	✔	✔	✔	✔	✔	✔	✔	✔	**2**
**H. Jiang (2013)**	✔	✗	✔	✔	✔	✔	✗	✔	✔	**2**
**E.Moon (2013)**	✔	✗	✔	✔	✔	✔	✗	✔	✔	**2**

**Y.Ogura (2013)**	✔	✔	✔	✔	✔	✔	✗	✗	✔	**2**
**J. Buchan (2014)**	✔	✗	✔	✔	✔	✔	✗	✔	✔	**2**
**T. Kotwicki (2014)**	✔	✗	✔	✔	✔	✔	✔	✔	✔	**2**

**B.Roye (2015)**	✔	✗	✔	✔	✔	✔	✔	✔	✔	**2**
**Q. Tang (2015)**	✔	✗	✔	✔	✔	✔	✗	✔	✔	**2**
**D.Bohl (2016)**	✔	✗	✔	✔	✔	✔	✔	✔	✔	**2**

**L. Xu (2016)**	✔	✗	✔	✔	✔	✔	✗	✔	✔	**2**
**Y. Ogura (2017)**	✔	✗	✔	✔	✔	✔	✗	✔	✔	**2**
**L.Xu (2017)**	✔	✗	✔	✔	✔	✔	✗	✔	✔	**2**

**Y. Takashi (2018)**	✔	✗	✔	✔	✔	✔	✗	✗	✔	**1**
**G. Man (2018)**	✔	✔	✔	✔	✔	✔	✔	✔	✔	**2**
**Y. Meng (2018)**	✔	✔	✔	✔	✔	✔	✔	✔	✔	**2**

**J. Zhang (2018)**	✔	✗	✔	✔	✔	✔	✗	✔	✔	**2**
**S. Mao (2018)**	✔	✗	✔	✔	✔	✔	✗	✔	✔	**2**
**F. Sheng (2018)**	✔	✔	✔	✔	✔	✔	✗	✔	✔	**2**

**B. Shi (2018)**	✔	✗	✔	✔	✔	✔	✗	✔	✔	**2**
**H. Jiang (2018)**	✔	✗	✔	✔	✔	✔	✗	✔	✔	**2**
**M. Andrusiewicz (2019)**	✔	✔	✔	✔	✔	✔	✔	✔	✔	**2**

**H. Jiang (2019)**	✔	✔	✔	✔	✔	✔	✗	✔	✔	**2**
**K.Takeda (2019)**	✔	✗	✔	✔	✔	✔	✗	✔	✔	**2**
**B. Shi (2019)**	✔	✗	✔	✔	✔	✔	✗	✔	✔	**2**

**Y.Wang (2020)**	✔	✗	✔	✔	✔	✔	✗	✔	✔	**2**
**K. Borysiak (2020)**	✔	✗	✔	✔	✔	✔	✔	✔	✔	**2**
**Y. Wang (2021)**	✔	✔	✔	✔	✔	✔	✗	✔	✔	**2**

**P. Carry (2021)**	✔	✗	✔	✔	✔	✔	✗	✔	✔	**2**
**P. Janusz (2021)**	✔	✔	✔	✔	✔	✔	✗	✔	✔	**2**
